# High Diversity of Glycosphingolipid Glycans of Colorectal Cancer Cell Lines Reflects the Cellular Differentiation Phenotype

**DOI:** 10.1016/j.mcpro.2022.100239

**Published:** 2022-04-28

**Authors:** Di Wang, Katarina Madunić, Tao Zhang, Oleg A. Mayboroda, Guinevere S.M. Lageveen-Kammeijer, Manfred Wuhrer

**Affiliations:** Leiden University Medical Center, Center for Proteomics and Metabolomics, RC Leiden, The Netherlands

**Keywords:** colorectal cancer, glycosphingolipids, glycosyltransferase, transcription factor, mass spectrometry porous graphitized carbon liquid chromatography, CMS, consensus molecular subgroup, CRC, colorectal cancer, EGCase I, endoglycoceramidase I, EMT, epithelial–mesenchymal transition, FBS, fetal bovine serum, GSL, glycosphingolipid, GT, glycosyltransferase, LC, liquid chromatography, MS, mass spectrometry, Neu5Gc, *N*-glycolylneuraminic acid, PCA, principal component analysis, PGC, porous graphitized carbon, RP, reverse phase, RT, room temperature, SPE, solid-phase extraction, TF, transcription factor

## Abstract

Colorectal cancer (CRC)–associated changes of protein glycosylation have been widely studied. In contrast, the expression of glycosphingolipid (GSL) patterns in CRC has, hitherto, remained largely unexplored. Even though GSLs are major carriers of cell surface carbohydrates, they are understudied due to their complexity and analytical challenges. In this study, we provide an in-depth analysis of GSL glycans of 22 CRC cell lines using porous graphitized carbon nano–liquid chromatography coupled with electrospray ionization–mass spectrometry. Our data revealed that the GSL expression varies among different cell line classifications, with undifferentiated cell lines showing high expression of blood group A, B, and H antigens while for colon-like cell lines the most prominent GSL glycans contained (sialyl)-Lewis^A/X^ and Lewis^B/Y^ antigens. Moreover, the GSL expression correlated with relevant glycosyltransferases that are involved in their biosynthesis as well as with transcription factors (TFs) implicated in colon differentiation. Additionally, correlations between certain glycosyltransferases and TFs at mRNA expression level were found, such as *FUT3*, which correlated with *CDX1*, *ETS2*, *HNF1A*, *HNF4A*, *MECOM,* and *MYB*. These TFs are upregulated in colon-like cell lines pointing to their potential role in regulating fucosylation during colon differentiation. In conclusion, our study reveals novel layers of potential GSL glycans regulation relevant for future research in colon differentiation and CRC.

## Highlights

• Undifferentiated cell lines showed high expression of blood group A, B, and H antigens.
• Colon-like cell lines are high in GSLs carrying (sialyl)-Lewis^A/X^ and Lewis^B/Y^ antigens.
• (Sialyl)-Lewis^A/X^ and Lewis^B/Y^ antigens associated with expression of *FUT3* and *CDX1.*
• I-branching was elevated in undifferentiated cells.

## Graphical Abstract

**Figure:**
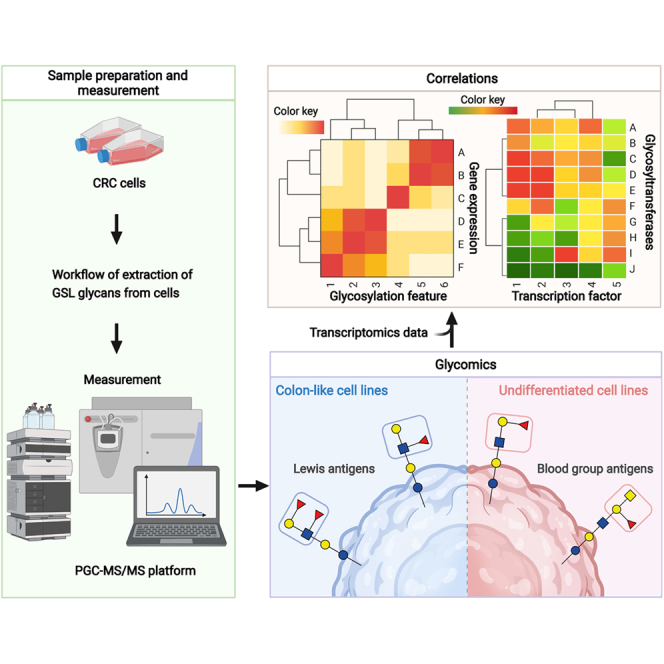

## In Brief

We performed an in-depth analysis of the GSL glycan profiles of 22 CRC cell lines revealing high expression of (sialyl)-Lewis^A/X^ and Lewis^B/Y^ antigens for colon-like cell lines while undifferentiated cell lines showed higher abundances of glycans with blood group A, B, and H antigens. Combining glycomics with transcriptomic data of relevant genes, significant correlations of Lewis structures with glycosyltransferase FUT3 and transcription factors were revealed, including CDX1.

Colorectal cancer (CRC) was the fourth most commonly diagnosed cancer (6.1%) and the second leading cause of cancer death (9.2%) worldwide in 2018 (1). Risk factors of CRC comprise genetics, aging, and lifestyle including low-fiber and high-fat diet, obesity, lack of physical exercise, as well as smoking (2). Currently, therapeutic solutions for individual CRC patients lack efficiency due to the heterogeneity of the disease, an asymptomatic clinical course as well as cancer diagnosis at late stages (3). Therefore, biomarker discovery is essential to allow early diagnosis and treatment of CRC and to gain a better understanding of the disease progression (4).

Various studies have shown that an altered glycosylation could be associated with the malignant transformation in colon cancer (4). Notably, CRC is characterized by a higher abundance of β1,6-branching as well as increased (poly-)*N*-acetyllactosamine extensions, (truncated) high-mannose types, and decreased bisected *N*-glycans. For the *O*-glycans, a higher expression of core 1 and core 2 *O*-glycans were found in CRC, an increase of (sialyl-)Tn-antigen, (sialyl-)T-antigen, as well as a decrease of core 3 and core 4 *O*-glycans. Moreover, sialyl-Lewis^A^ has been found to be highly expressed in the colon and rectum during neoplastic transformation as well as cancer progression. This antigen is known to be involved in the adhesion of cancer cells to E-selectin present on endothelial cells, playing an important role in the metastasis of the colon cancer cells (5). In addition, sialyl-Lewis^A^ has been correlated with a worse prognosis within each stage and is considered as a prognostic factor for patients with advanced CRC (6, 7).

Glycosphingolipids (GSLs) play essential roles in various biological processes including cellular development, differentiation, and cell fate decisions (8). Taking this into account, it is not surprising that alterations of the GSL glycosylation have been found for malignant transformation including a decrease of disialylated gangliosides and globosides (except for Gb3 [Gal*α*1,4Galβ1,4Glcβ1ceramide]) (4, 9). Another study revealed specific changes of GSL glycans in CRC tumor tissue compared with controls, including the increase of fucosylation and the reduction of acetylation, sulfation, globosides, and disialylgangliosides (10). Misonou *et al.*, found enhanced fucosylation and sialylation of GSLs in CRC tumors when compared to control tissues (11). Gangliosides are involved in the regulation of cell adhesion and motility, further resulting in the initiation of metastasis in many cancers (12). In addition, Gb3 and certain gangliosides, such as GD3 (NeuAc*α*2,8NeuAc*α*2,3Galβ1,4Glcβ1ceramide) and GM2 (GalNAcβ1,4[NeuAc*α*2,3]Galβ1,4Glcβ1ceramide), contribute to the tumor-associated angiogenesis (12, 13, 14). Moreover, enhanced expression of Gb3 has been found in colorectal adenocarcinomas and their metastases in comparison with normal tissue (15, 16).

Importantly, CRC cell lines are widely used model systems as they recapitulate the molecular alterations and genomic profiles of primary tumors as demonstrated by genomic studies, drug sensitivity screening, as well as DNA-, RNA-, and protein-profiling (17, 18, 19). Previously, 34 CRC cell lines have been studied based on integrated data analysis of mutations, RNA, and protein expression (19). The cell lines were grouped into two distinct clusters, the colon-like cell lines expressed gastrointestinal differentiation markers and undifferentiated cell lines showed an upregulation of epithelial–mesenchymal transition (EMT) and transforming growth factor β signaling (19). Moreover, the analysis of the *O*-glycome of 26 CRC cell lines revealed a high abundance of I-branched and (sialyl-)Lewis^A/X^ antigens for colon-like cell lines while undifferentiated cell lines were dominated by truncated *α*2,6-core sialylated glycans in the absence of Lewis structures (20).

To date, there is only limited information on the expression and regulation of GSL glycans in CRC cell lines; thus, we performed an in-depth analysis of GSL glycans of 22 CRC cell lines to investigate the GSL glycan expression. For this analysis, the GSL glycan head groups were enzymatically released from their lipid portion, followed by analysis using porous graphitized carbon (PGC) nano–liquid chromatography (LC) electrospray ionization tandem mass spectrometry (MS) in negative ionization mode. Associations between glycosylation features and gene expression levels of glycosyltransferases (GTs) and transcription factors (TFs) were explored, shedding light onto the potential regulation of glycosylation pathways in colon differentiation.

## Experimental Procedures

### Materials

TFA and chloroform were obtained from Merck. Methanol (MeOH) of ultra LC-MS grade was purchased from Actuall Chemical. NaBH_4_, HCl, ammonium bicarbonate, and Dowex cation-exchange resin (50W-X8) were obtained from Sigma–Aldrich. KOH and 100% glacial acetic acid were from Honeywell Fluka. C18 Ziptips were purchased from Millipore and TopTip (microspin column; empty column) from Glygen Corporation. Reverse phase (RP) tC18 solid-phase extraction (SPE) cartridges (50 mg) were purchased from Waters, and SPE bulk sorbent Carbograph was from BGB Analytik USA LLC. 2-Propanol was obtained from Biosolve Chimie. Acetonitrile (MeCN) of LC-MS grade was from Biosolve. Endoglycoceramidase I (EGCase I), *α*1-3,4 fucosidase, *α*1-2,4,6 fucosidase O, *α*2-3 neuraminidase S, purified bovine serum albumin, 10 × GlycoBuffer 1, and 1 × EGCase I reaction buffer were purchased from New England Biolabs. Dulbecco’s modified Eagle’s medium (DMEM) and Hepes-buffered RPMI1640 medium were purchased from Gibco (Thermo Fisher Scientific). Fetal bovine serum (FBS) was from Bodinco BV. Penicillin/streptomycin from Invitrogen, 0.5% trypsin-EDTA solution 10× was ordered from Santa Cruz Biotechnology, and T75 cell culture flasks were obtained from Greiner-Bio One B.V. Ultrapure water generated by an ELGA PURELAB system was used for all solvent preparations and washing steps.

### Cell Lines and Cell Culture

The human CRC cell lines were provided by the Department of Surgery and the Department of Cell and Chemical Biology of Leiden University Medical Center as well as the Department of Molecular Cell Biology and Immunology of Amsterdam UMC. Cells were cultured in RPMI1640 medium, except for cell lines LS174T, SW1398, and SW948, which were cultured with DMEM. All media were supplemented with 10% FBS and 1% antibiotics [streptomycin (5 mg/ml) and penicillin (5000 IU/ml)]. Cultures were performed in a cell incubator with 5% CO_2_ in humidified air at 37 °C. A trypsin/EDTA solution was used to detach the cells when approximately 80% confluence was reached. The medium was used to terminate the enzymatic activity. The harvested cells were counted with a TC20 automated cell counter. The cells pellets were obtained by centrifuging at 1500*g* for 5 min after being washed twice with 1 × PBS. The prepared cell pellets were stored at −20 °C. All CRC cell lines have been tested by means of the Promega PowerPlex Fusion System 5C autosomal STR kit. More details about cell culturing, cell origin, and the characteristics of the cell lines are provided in supplemental Table S-1.

### Extraction and Purification of GSLs From Cells

A detailed procedure for the extraction and purification of GSLs from cells was described in a previous study (21). In brief, two million cells were lysed in 200 μl of H_2_O, followed by the addition of 500 μl of chloroform and 350 μl of MeOH; after each addition the plate was shaken on a horizontal shaker for 10 min, followed by sonication for 10 min. Afterward, the sample was shaken for 4 h at room temperature (RT), followed by centrifugation for 15 min at 3000*g* at 20 °C. A 400 μl mixture of chloroform/MeOH (2:1; *v/v*) and 400 μl of MeOH/H_2_O (1:1; *v/v*) were added sequentially to the sample after collection of the upper phase. The sample mixture was shaken overnight at 1000 rpm at RT followed by the collection of the upper phase. Another two extractions were performed after adding 400 μl of MeOH/H_2_O (1:1; *v/v*) to the sample, followed by a centrifugation step (15 min at 3000*g* at 20 °C). The collected upper phase was combined into a single glass vial and dried under a vacuum. The dried sample was dissolved in 100 μl of MeOH and incubated for 30 min on a shaker, followed by the addition of 100 μl of H_2_O to the suspended sample. The sample was loaded onto the tC18 RP SPE cartridge, which was preconditioned with the sequential addition of 1 ml chloroform/MeOH (2:1; *v/v*), 1 ml MeOH, and 2 ml MeOH/H_2_O (1:1; *v/v*). The loaded cartridge was washed using 2 ml of MeOH/H_2_O (1:1; *v/v*), and elution was performed by sequentially adding 2 ml of MeOH and 2 ml of chloroform/MeOH (2:1; *v/v*). The combined eluate was dried under vacuum.

### Enzymatic Release and Purification of GSL Glycans

The GSL glycan head groups were enzymatically released from extracted GSLs on the basis of the manufacturer’s instruction. In brief, the purified GSLs were suspended with 36 μl of H_2_O, 4 μl EGCase I reaction buffer, and 2 μl EGCase I enzyme. The reaction system (42 μl) was incubated at 37 °C for 36 h. The purification of released glycans was performed on tC18 RP SPE cartridge preconditioned with 2 ml of MeOH and 2 ml of H_2_O. The loaded cartridge was washed twice with 200 μl of H_2_O. A sample mixture that contained the flowthrough and the solution after washing was collected and was lyophilized under a vacuum.

### Reduction and Purification of Reduced GSL Glycan Alditols

The purified GSL glycans were dissolved in 40 μl of freshly prepared 1 M NaBH_4_ in 50 mM KOH, followed by incubation at 50 °C for 3 h. The reaction was quenched using 4 μl of glacial acetic acid. The mixture was loaded onto the RP C18 Ziptips containing 50W-X8 resin, which was preconditioned with 3 × 60 μl of 1 M HCl, 3 × 60 μl of MeOH, and 3 × 60 μl of H_2_O. The loaded cartridge was washed twice with 40 μl of H_2_O. The flowthrough and washing were collected and dried under a vacuum. To remove residual borate, 150 μl of MeOH was added twice during the lyophilization process.

PGC cleanup was performed as described in a previous study (22). Briefly, the dried reduced sample was resuspended in 40 μl of H_2_O with 0.1% TFA (*v/v*) with shaking at RT. The empty TopTip containing 50 μl of PGC slurry was preconditioned sequentially with 60 μl of MeOH, 3 × 60 μl of 80% MeCN with 0.1% TFA (*v/v*), and 3 × 60 μl of H_2_O with 0.1% TFA (*v/v*), followed by sample loading. The loaded column was washed with 2 × 60 μl of H_2_O with 0.1% TFA (*v/v*). The sample was eluted with 2 × 40 μl of 60% MeCN with 0.1% TFA (*v/v*); after pooling the two eluates, the sample was dried under vacuum.

### Measurement Using PGC-nanoLC-MS/MS

The purified GSL glycans alditols were reconstituted in 20 μl of H_2_O. A fraction of the sample (2 μl) was loaded onto a Hypercarb PGC trap column (5 μm Hypercarb Kappa, 320 μm × 30 mm, in-house packed) and separated on a Hypercarb PGC nano-column (3 μm Hypercarb Kappa, 75 μm × 100 mm, in-house packed) within a Dionex Ultimate 3000 nanoLC system and analyzed with an amaZon speed ion trap MS (Bruker Daltonics). Solvent A consisted out of 10 mM ammonium bicarbonate and solvent B out of 60% MeCN in 10 mM ammonium bicarbonate. The separation was performed over a linear nanoLC gradient of solvent B ranging from 1% up to 65% in 80 min with flow rate of 0.5 μl/min. The column was washed with 95% solvent B for 10 min. The GSL glycans were analyzed with capillary voltage of 1000 V, 280 °C of drying gas at 5 l/min, and the nebulizer was set at 3 psi. To enhance sensitivity, 2-propanol enriched nitrogen gas was used for enhancing sensitivity (23). MS spectra were obtained within the *m/z* range 340 to 2000 in negative ion mode with *m/z* 1200 as the target mass of the smart parameter settings. MS/MS spectra were produced by collision-induced dissociation on the top three abundance precursors.

### Exoglycosidase Digestion

Prior to exoglycosidase digestions, a pooled sample was generated by combining 30 μl of each cell line sample. As a positive control, GSL glycans released from two million PaTu-S cells was used (21). The reaction was performed according to the manufacturer’s instructions. Briefly, the corresponding reagents and samples were added into the reaction system and incubated at 37 °C overnight, followed by purification and drying under vacuum. More details about each exoglycosidase experiment can be found in supplemental Table S-2.

### Data Analysis

Structural assignments were based on accurate mass, elution properties during the separation on a PGC-nanoLC column, the manual inspection of MS/MS spectra according to previously identified diagnostic ions in negative mode for GSLs, exoglycosidase digestion, as well as known biosynthetic pathways of GSL glycans (24, 25) https://rb.gy/mt4miw (accessed 10 May) (26, 27). Bruker Data analysis software (version 5.0) was used for data analysis. In detail, extracted ion chromatograms were generated by extracting the theoretical mass of the first three isotopes of GSL glycans of the observed (singly and doubly charged) species. The peak area under the curve was generated by integrating each peak, which had a signal to noise ratio ≥6 for all the technical and biological replicates. Relative quantification was conducted on the total area of all GSL glycans detected in one sample that was normalized to 100%. Further data analysis and visualization were performed in “R” software (version 4.0.5; https://cran.r-project.org/bin/windows/base/old/4.0.5/) using packages “tidyverse,” “Rcpm,” “pcaMethods,” “stringi,” “readxl,” “ggplot2,” “ggrepel,” “reshape2,” "ggpubr," “tidyHeatmap,” “corrplot,” “ComplexUpset,” and “data.table,”

### Experimental Design and Statistical Rationale

We performed an in-depth analysis of GSL glycans in 22 CRC cell lines by PGC-nanoLC-electrospray ionization-MS/MS in negative ionization mode. Full technical replicates were performed for all CRC cell lines. In addition, for the cell lines Colo320 and HCT15, biological replicates were performed (*i.e.*, analyses of independent cell cultures). More information about the technical replicates of each sample is given in supplemental Tables S-3.1- S-3.22. The minimum positive number (0.00001) as an imputation was used for the application of statistical tools such as principal component analysis (PCA), which are sensitive to the missing values. Spearman correlations were assessed between the GSL glycan data of this study and published transcriptomics data of the same cell lines obtained from public repositories (19).

## Results

### Striking Differences in GSL Glycan Expression in CRC Cell Lines

GSLs were extracted from harvested cells, followed by enzymatic release of the GSL glycan head groups. In total, 94 GSL glycans could be structurally identified in CRC cell lines with sizes varying from 3 up to 12 monosaccharides (supplemental Table S-4). The identified GSL glycans were grouped on the basis of glycan structural features and a highly diverse GSL glycosylation was observed for the different CRC cell lines (supplemental Fig. S-1; supplemental Tables S-3.1- S-3.22 and S-5).

**Fig. 1:**
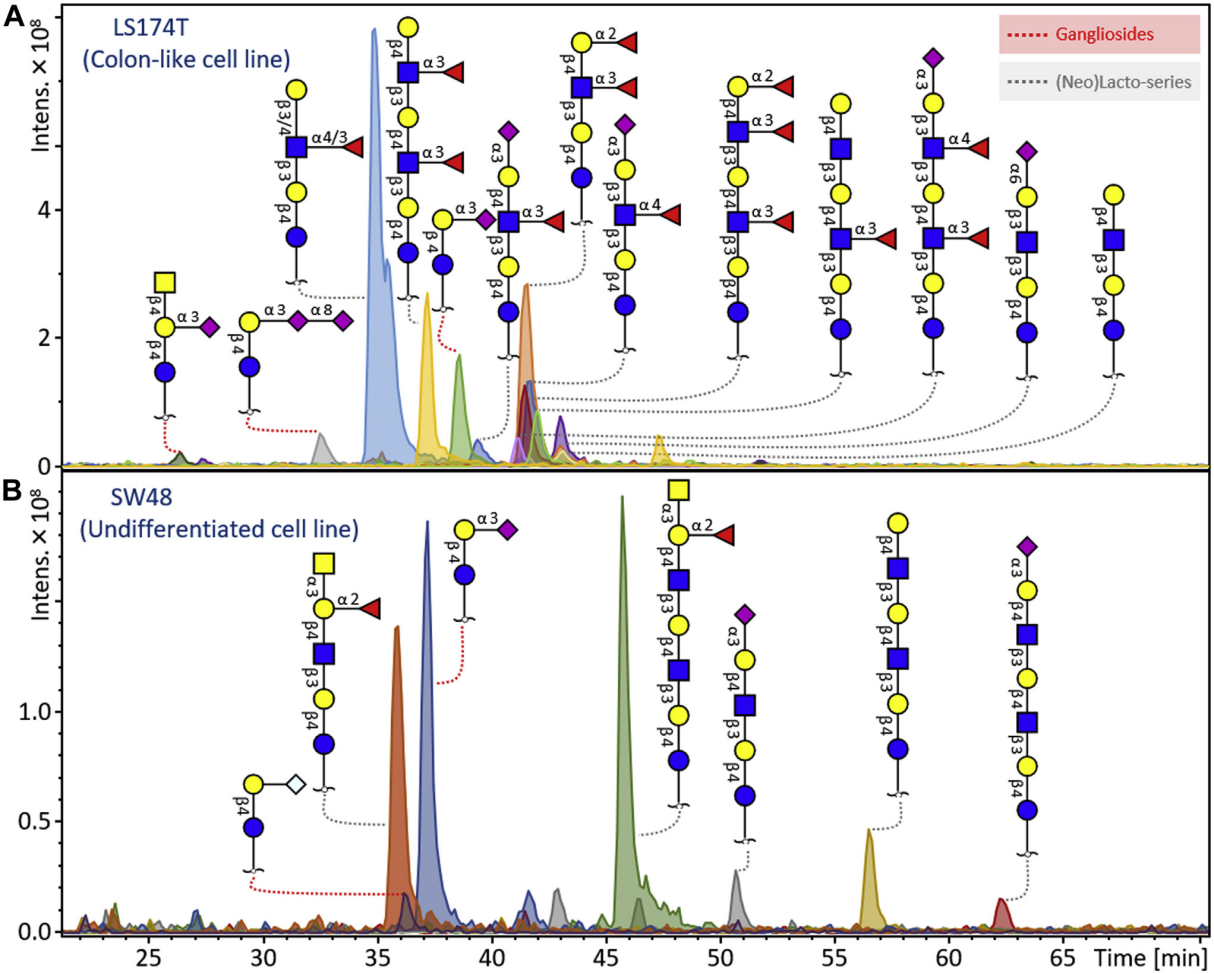
**GSL glycans profile of two CRC cell lines.***A*, the GSL glycans profile of the colon-like cell line LS174T displays a high expression of (sialyl-)Lewis–type antigens on (neo)lacto-series GSLs. *B*, undifferentiated human colon adenocarcinoma cell line SW48 is mainly characterized by the high expression of blood group A antigens and ganglioside GM3. CRC, colorectal cancer; GSL, glycosphingolipid.

The diversity of GSL glycan expression for two representative CRC cell lines is exemplified in Figure 1. The colon-like cell line LS174T was mainly characterized by a relatively high expression of a diverse set of (neo)lacto-series GSL glycans with (sialyl-)Lewis–type antigens (see Table 1 for all the specific GSL glycosylation features). In contrast, the undifferentiated cell line SW48 showed high levels of (neo)lacto-series GSL glycans with blood group A antigens and GM3 ganglioside structures (supplemental Table S-5). The overall GSL glycan repertoire of the 22 CRC cell lines revealed that blood group antigens A, B, and H were mainly found on (neo)lacto-series glycans, whereas blood group H was also present on some ganglioside and globo-series glycans. Additionally, cell line SW480 was the only cell line that contained a globo-series glycan with blood group A (relative abundance of 4%).

**Table 1:** GSL glycosylation features, structure, and average relative abundances for different CRC cell line classifications

Glycosylation feature	Structure	Average relative abundance (%) in colon-like cell lines		Average relative abundance (%) in undifferentiated cell lines	
Fucosylation^a^		105	↑	41	↓
*α*1,2-fucosylation		38	↑	37	↓
*α*1,3/4-fucosylation^a^		66	↑	4	↓
Sialylation^b^		39	↓	53	↑
*α*2,3-sialylation^b^		32	↓	44	↑
α2,6-sialylation		4	↓	5	↑
LewisA/X^a^		19	↑	0.2	↓
LewisB/Y^a^		20	↑	4	↓
Sialyl-LewisA/X^a^		9	↑	0.4	↓
A-Lewis^B/Y^^a^		8	↑	0	↓
Blood group A^b^		7	↓	13	↑
Blood group B^b^		0	↓	4	↑
Blood group H^b^		3	↓	1	↑
I-branching^b^		0	↓	1	↑

Arrows indicate relatively high and low levels of glycosylation features in colon-like *versus* undifferentiated CRC cell lines.

a glycosylation features with relatively higher expression in colon-like cell lines.

b glycosylation features with higher abundance in undifferentiated cell lines.

Gangliosides were expressed in all CRC cell lines with various abundances, ranging from 0.2% in colon-like cell line SW1463 up to 100% in the neuroendocrine carcinoma-derived cell line Colo320 (unassigned cell line) (supplemental Table S-6). GM3 was the main ganglioside detected in most cell lines except for SW620 and SW1463 (supplemental Table S-5). Apart from cell line Colo320, all cell lines showed expression of (neo)lacto-series glycans, of which the SW1463 cell line had the highest relative abundance with 98% (supplemental Table S-6). The highest abundance of globo-series glycans was detected in SW480 cell line with a relative abundance of 17%. From the 94 detected GSL glycans in this study, only 27 were expressed in all CRC cell line classifications (colon-like, undifferentiated, and unassigned; supplemental Fig. S-2 and Table S-7). Many GSL glycans were found to be unique for a specific CRC cell line classification (supplemental Fig. S-2 and Table S-7). Specifically, 23 (neo)lacto-series glycans were only expressed in colon-like cell lines showing glycosylation features such as (A-)Lewis^B/Y^, blood group H, and (sialyl-)Lewis^A/X^ antigens. The undifferentiated cell lines exhibited 20 unique GSL glycans, with glycosylation features such as blood groups A, B, and H as well as I-branched glycans. Only three GSL glycans were found only in the unassigned cell lines, all belonging to the (neo)lacto-series with B-Lewis^B/Y^, blood group B, and I-branched antigens.

### Association of Glycosylation Features With Cell Line Differentiation

**Fig. 2:**
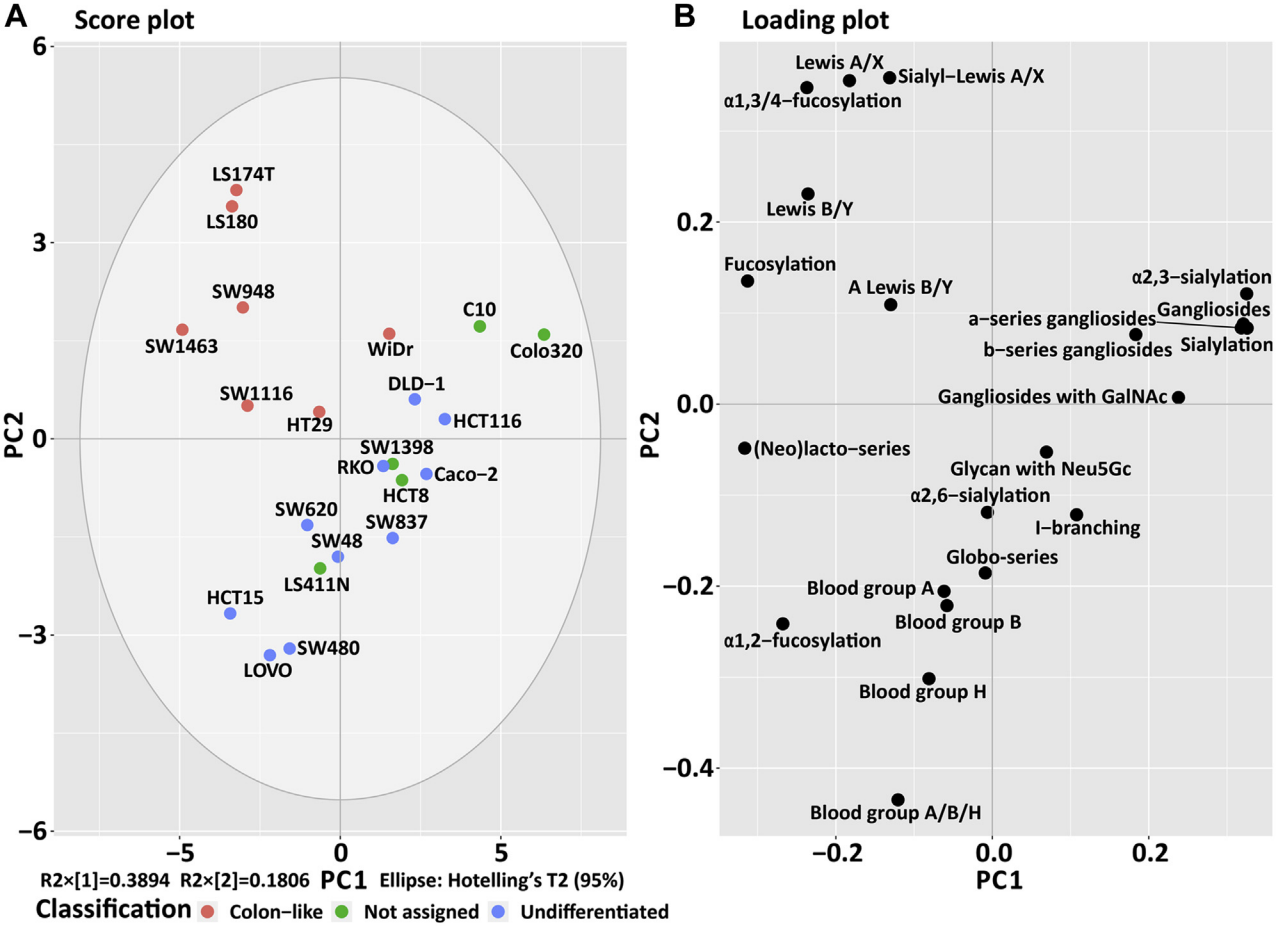
**PCA of glycosylation features of CRC cell lines.** (*A*) PCA score plot and (*B*) loadings plot of PC1 against PC2. The first two principal components explain 57% of the variation within the data. Technical replicates were averaged per cell line. CRC, colorectal cancer; PC, principal component; PCA, principal component analysis.

To further investigate the association of specific GSL glycosylation features with the CRC classifications, a PCA was applied (Fig. 2 and supplemental Table S-6). In order to address the sparsity of individual GSL glycans detected in this study, and to account for the shared biosynthetic steps between them, GSL glycans were grouped into gangliosides (a-series and b-series), (neo)lacto-series and globo-series, and further categories were established on the basis of the expression of structural motifs (Table 1), for example, total-, *α*1,2-, and *α*1,3/4-fucosylation; total-, *α*2,3-, and *α*2,6-sialylation; (sialyl-)Lewis^A/X^, and (A) Lewis^B/Y^; and blood group A, B, H, as well as I-branching. The analyzed 22 CRC cell lines were classified into two groups based on colon cell differentiation: colon-like cell lines (7) and undifferentiated cell lines (10), and the remaining cell lines were unassigned (5) as there was no conclusive data available that would have allowed their assignment (19).

A score plot of the PCA model built on the glycosylation features showed a clear clustering according to cell line differentiation (Fig. 2). The colon-like cell lines LS174T, LS180, SW948, SW1463, and SW1116 (Fig. 2*A*) mainly grouped in the *top left* of the score plot and showed a higher expression of glycans with (sialyl-)Lewis^A/X^ and (A-)Lewis^B/Y^ antigens, as well as (*α*1,3/4-)fucosylation (Fig. 2*B*). In all colon-like cell lines sialyl-Lewis^A/X^ antigens were detected at high abundance. Specifically, mucin-secreting cell lines LS180 and LS174T were characterized by the very high expression of (sialyl-)Lewis^A/X^ and *α*1,3/4-fucosylation. Lewis^B/Y^ antigens showed highest expression in colon-like cell line SW948 while A-Lewis^B/Y^ antigens were found to have the highest expression in colon-like cell line SW1463 (supplemental Fig. S-1 and Table S-8).

In contrast, undifferentiated cell lines SW620, SW48, HCT15, LOVO, SW480, and the unassigned cell line LS411N showed high expression of globo-series glycans and A, B, and H blood group antigens. The highest expression of blood group A and H antigens was detected in the undifferentiated cell lines SW48 (50%) and HCT15 (55%), respectively (supplemental Fig. S-1 and Table S-8). Only two cell lines (LS411N and LOVO) were found to express GSLs that carry blood group B antigens (23% and 35%, respectively). I-branching played an important role in the clustering of unassigned cell lines HCT8 (2%) and SW1398 (3%) as well as undifferentiated cell lines Caco-2 (5%) and SW837 (9%) (Fig. 2; supplemental Fig. S-1 and Table S-8).

In addition, glycans with *N*-glycolylneuraminic acid (Neu5Gc) were found in most of the undifferentiated cell lines with various abundances ranging from 0.2% (SW837) up to 5% (RKO) (supplemental Fig. S-1 and Table S-8). It is worth noting that cell lines clustered well based upon consensus molecular subgroup (CMS) classification (28) and their expression of GSL glycans (supplemental Fig. S-3).

The clustering of CRC cell lines C10, Colo320, WiDr, DLD-1, and HCT116 (Fig. 2*A*) was driven by a high expression of (a- and b-series) gangliosides, gangliosides with GalNAc, and (*α*2,3-)sialylation (*right part* of the loading plot of Fig. 2*B*). Gangliosides and specifically a-series gangliosides were highly expressed in the unassigned cell lines HCT8, SW1398, C10, and Colo320 as well as undifferentiated cell lines Caco-2, DLD-1, HCT116, and one colon-like cell line (WiDr). A high abundance of b-series gangliosides was detected in undifferentiated cell lines RKO and SW837 as well as for the unassigned cell lines Colo320 and C10. Gangliosides with GalNAc were highly expressed in undifferentiated cell lines SW837, Caco-2, and HCT116 as well as unassigned cell lines SW1398 and Colo320. The high expression of *α*2,3-sialylation, overall sialylation, and gangliosides was observed in undifferentiated cell lines Caco-2, DLD-1, and HCT116, colon-like cell line WiDr, as well as unassigned cell lines HCT8, SW1398, C10, and Colo320. On the other hand, globo-series glycans were highly abundant in undifferentiated cell lines HCT15, SW480, Caco-2, HCT116, and colon-like cell line SW1116 as well as the unassigned cell line HCT8. The findings and relative quantification of GSL glycans in CRC cell lines are summarized in supplemental Fig. S-1 and Table S-8.

### The Correlation Between Glycosyltransferases and GSL Glycans Expression

**Fig. 3:**
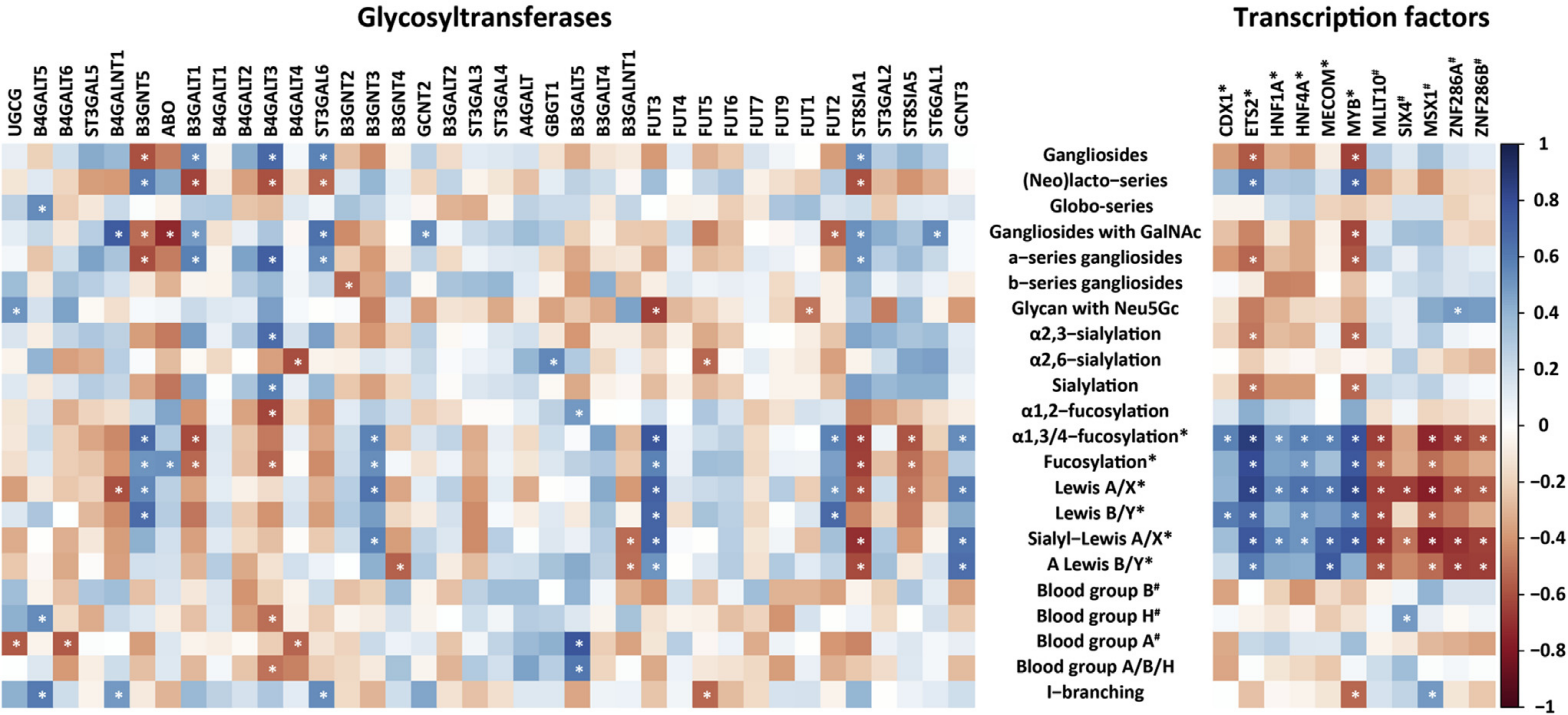
**Correlation matrix for glycosylation features of CRC cell lines and the expression of genes encoding relevant GTs (*left*) and TFs (*right*).** Glycosylation traits and TFs marked with ∗ show higher expression in colon-like cell lines, whilst those with # display higher abundance in undifferentiated cell lines. Spearman correlation was performed with the significance cut-off *p* < 0.05. *White asterisks* mark statistically significant correlations. The correlation coefficients are color coded as indicated in the right key bar. CRC, colorectal cancer; GT, glycosyltransferase; TF, transcription factor.

To get further insights into the glycosylation phenotypes, a correlation analysis was performed between the gene expression of GTs and expression of glycosylation features presented in CRC cell lines. A dataset containing mRNA expression of relevant GTs involved in the biosynthesis of GSL glycans was obtained from a previous study (19). The correlations between GSL glycosylation and relevant GTs are shown in Figure 3. Although it did not meet the significance threshold, a trend toward a positive correlation was found between *ST3GAL5* and the expression of gangliosides (r = 0.44, *p* = 0.08), specifically the a-series gangliosides (r = 0.46, *p* = 0.06). The gene *B4GALNT1*, which encodes the corresponding GT responsible for adding GalNAc to Gal in ganglioside biosynthesis, was positively correlated with gangliosides containing a GalNAc (ganglioside with GalNAc, r = 0.73, *p* = 0.001). Moreover, *ST8SIA1*, whose corresponding GT is responsible for the biosynthesis of b-series gangliosides, positively associated with ganglioside expression (r = 0.54, *p* = 0.03). In addition, the expression of (neo)lacto-series glycans showed positive correlation with *B3GNT5* (r = 0.60, *p* = 0.01), which encodes the key enzyme for the biosynthesis of (neo)lacto-series glycans. No significant correlation was found between globo-series glycans and *A4GALT*, which encodes the GT that catalyzed the initial step of their biosynthesis.

Further correlations were found between several terminal glycosylation motifs and responsible transferases. For example, *α*2,3-sialylation showed positive correlation with *ST3GAL6*, which encodes the enzyme responsible for adding the *α*2,3-linked sialic acid. Additionally, *ST6GAL1* encoding the GT, which adds a sialic acid in *α*2,6-linkage, showed a trend toward a positive correlation with *α*2,6-sialylation. *FUT3* expression had significant positive correlations with fucosylation, *α*3,4-fucosylation, Lewis^A/X^, sialyl-Lewis^A/X^, Lewis^B/Y^, and A Lewis^B/Y^. *FUT2* showed significant positive correlation with Lewis^B/Y^ antigen expression. Moreover, positive correlations were found between B3GNT5 and *α*3,4-fucosylation, fucosylation, Lewis^A/X^, and Lewis^B/Y^. *GCNT2* encoding a GT responsible for synthesis of I-branched glycans showed a trend toward a positive correlation with I-branched glycans (r = 0.45, *p* = 0.07), although it did not meet the significance threshold. All significant r-values are indicated in Table 2, for all r- and *p*-values see supplemental Table S-9.

**Table 2:** Correlation between GSL glycans and expression of relevant glycosyltransferases and selected transcription factors

Subgroups and derived traits of GSL glycans	Glycosyltransferase									Transcription factor										
	ST3GAL5	B4GALNT1	B3GNT5	ST3GAL6	GCNT2	FUT3	FUT2	ST8SIA1	ST6GAL1	CDX1^a^	ETS2^a^	HNF1A^a^	HNF4A^a^	MECOM^a^	MYB^a^	MLLT10^b^	SIX4^b^	MSX1^b^	ZNF286A^b^	ZNF286B^b^
Gangliosides	0.44							0.54												
(Neo)lacto-series			0.60																	
Gangliosides with GalNAc		0.73																		
a-series gangliosides	0.46																			
α2,3-sialylation				0.48																
α2,6-sialylation									0.47											
α1,3/4-fucosylation			0.67			0.77				0.57	0.86	0.49	0.58	0.58	0.78	−0.65	−0.34	−0.73	−0.64	−0.58
Fucosylation			0.51			0.58				0.41	0.80	0.47	0.49	0.34	0.76	−0.52	−0.32	−0.50	−0.42	−0.33
Lewis^A/X^			0.55			0.71				0.41	0.83	0.55	0.64	0.61	0.83	−0.65	−0.65	−0.76	−0.60	−0.57
Lewis^B/Y^			0.66			0.70	0.67			0.60	0.67	0.40	0.52	0.47	0.60	−0.63	−0.17	−0.55	−0.48	−0.33
Sialyl-Lewis^A/X^						0.69				0.36	0.75	0.56	0.51	0.70	0.76	−0.67	−0.50	−0.76	−0.69	−0.65
A Lewis^B/Y^						0.51				0.20	0.61	0.43	0.42	0.74	0.42	−0.58	−0.43	−0.52	−0.67	−0.66
Blood group B																		0.41		
Blood group H																	0.50			
I-branching					0.45															

Spearman coefficients (r) are given for significant correlations (*p* < 0.05).

a The transcription factors are upregulated in colon-like cell lines.

b The transcription factors are upregulated in undifferentiated cell lines.

### The Association of Expression of GSL Glycans With TFs

A recent study revealed that gene expression of TFs *CDX1*, *ETS2*, *HNF1A*, *HNF4A*, *MECOM*, and *MYB* showed higher expression in colon-like cell lines in comparison to undifferentiated cell lines, which had higher expression of *MLLT10*, *MSX1*, *SIX4*, *ZNF286A*, and *ZNF286B* (20). To get further insights into the potential regulation of GSLs expression in CRC cell lines, associations of glycosylation features with TFs were evaluated (Fig. 3). Significant correlations were found between glycosylation features and several TFs. The glycosylation feature *α*1,3/4-fucosylation, which was high in colon-like cell lines, showed a positive correlation with *CDX1*, *ETS2*, *HNF1A*, *HNF4A*, *MECOM*, and *MYB*, whereas, it showed negative correlations with TFs *MLLT10*, *MSX1*, *ZNF286A*, and *ZNF286B*. Significant correlation was found for Lewis^A/X^ with *ETS2*, *HNF1A*, *HNF4A*, *MECOM*, *MYB*, *MLLT10*, *SIX4*, *MSX1*, *ZNF286A*, and *ZNF286B*. Sialyl-Lewis^A/X^ correlated positively with ETS2, HNF1A, *HNF4A*, *MECOM*, and *MYB* but negatively with *MLLT10*, *SIX4*, *MSX1*, *ZNF286A*, and *ZNF286B*. Lewis^B/Y^ showed positive correlations with *CDX1*, *ETS2*, *HNF4A*, *MYB*, *MLLT10*, and *MSX1*. A Lewis^B/Y^ correlated positively with *ETS2* and *MECOM* and negatively with *MLLT10*, *MSX1*, *ZNF286A*, and *ZNF286B*. Additionally, a positive correlation of (neo)lacto-series was found with *ETS2* and *MYB*. Meanwhile, *SIX4* showed high expression in undifferentiated cell lines and revealed a positive correlation with blood group H that was highly expressed in undifferentiated cell lines (Fig. 2 and supplemental Fig. S-1). Additionally, *MSX1* showed a trend toward positive correlation with blood group B (r = 0.41, *p* = 0.10), which was also highly expressed in the undifferentiated cell line LOVO (supplemental Fig. S-1). All significant r-values are presented in Table 2; for all r- and *p*-values see supplemental Table S-9.

In order to investigate the role of TFs in relation to GTs, a correlation analysis was performed between TFs and GTs that are known to be involved in the biosynthesis of GSL glycans. *FUT3*, which showed positive correlations with Lewis antigens expression and *α*1,3/4-fucosylation, correlated positively with *CDX1*, *ETS2*, *HNF1A*, *HNF4A*, and *MYB*, which are all highly expressed in colon-like cell lines (supplemental Fig. S-4). *B3GNT5* showed positive correlation with TFs that were highly expressed in colon-like cell lines and presented a negative correlation with the TFs that showed high expression in the undifferentiated cell lines. *B4GALNT1*, encoding a key GT for gangliosides series, showed positive correlations with *SIX4* and *MSX1*, both highly expressed in undifferentiated cell lines. Additionally, *ST8SIA1* revealed a negative association with *ETS2*, *HNF1A*, *MECOM*, and *MYB* and correlated positively with *MLLT10*, *SIX4*, *MSX1*, *ZNF286A*, and *ZNF286B*, which were highly expressed in the undifferentiated cell lines. All *p*- and r-values can be found in supplemental Table S-10.

### The Association of GSL Glycan and O-glycan in CRC Cell Lines

We recently performed an in-depth *O*-glycosylation analysis of different CRC cell lines (20) which allowed us to compare the expression patterns of glycosylation features shared between *O*-glycans and GSL glycans. Positive correlations between the same glycosylation features of the GSL glycome and the *O*-glycome (supplemental Fig. S-5 and Table S-11) included Lewis^A/X^, sialyl-Lewis^A/X^, blood group B, blood group H, and *α*2,6-sialylation. Moreover, the Lewis^A/X^ antigens of the GSL glycome showed significant positive correlations with specific *O*-glycan features such as sialyl-Lewis^A/X^ structures, sulfo-Lewis^A/X^, and (sialyl-)dimeric Lewis^A/X^. Similarly, the sialyl-Lewis^A/X^ (GSL glycome) had positive correlations with the additional *O*-glycan features such as Lewis^A/X^, sulfo-Lewis^A/X^, and (sialyl-)dimeric Lewis^A/X^. All *p*- and r-values can be found in supplemental Table S-11.

## Discussion

In this study, the GSL head groups of 22 CRC cell lines were investigated using a PGC-nanoLC-MS/MS platform, showing a highly diverse GSL glycome. The correlations of specific glycosylation features were investigated with corresponding GTs involved in the biosynthesis of GSL glycans as well as with certain TFs. In addition, this study revealed the associations between TFs and GTs related to the GSL biosynthetic pathway.

**Fig. 4:**
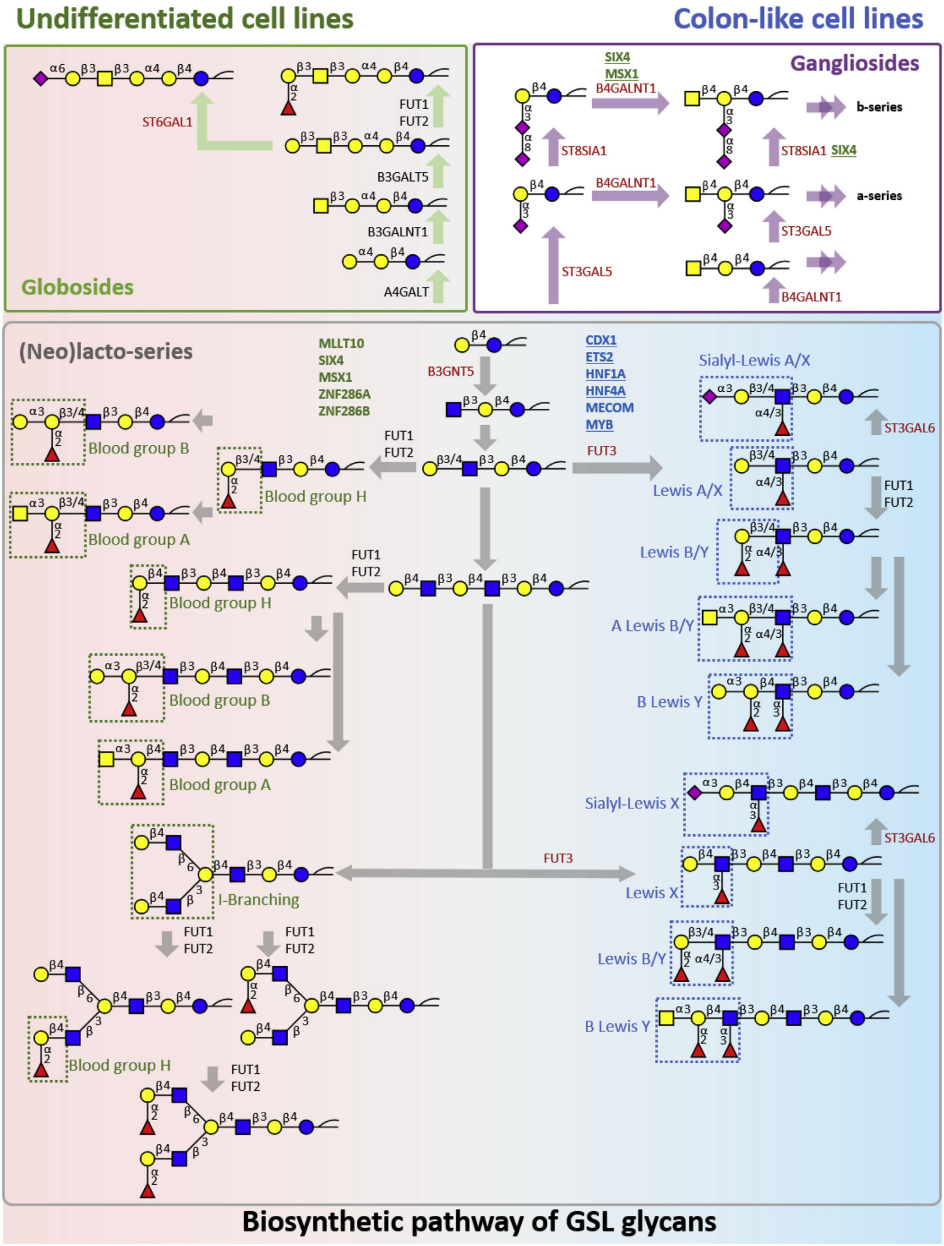
**Differences in the expression of GSL glycans between colon-like and undifferentiated CRC cell lines including the relevant genes involved in the biosynthetic pathway.** The main subgroups of GSL glycans globo-series, gangliosides, and (neo)lacto-series are presented in *green*, *purple*, and *gray rectangles*, respectively. GTs that correlated positively with corresponding glycosylation features or subgroups of GSL glycome are marked in *red*. TFs upregulated in colon-like cell lines or undifferentiated cell lines are indicated in *bold blue* or *bold green*, respectively. Underlined TFs indicate a significant positive correlation with the expression of certain GTs. *Dashed green* and *blue rectangles* indicate the high expression of corresponding glycosylation feature in undifferentiated cell lines and colon-like cell lines, respectively. The display of gangliosides on a *white background* indicates a similar expression for both classifications. *Double arrows* display that glycans can be further elongated. CRC, colorectal cancer; GSL, glycosphingolipid; GT, glycosyltransferase; TF, transcription factor.

The differences in the expression of GSL glycome, relevant GTs, and TFs of colon-like and undifferentiated CRC cell lines can be interpreted on the basis of the known GSL biosynthetic pathway (Fig. 4). Overall, blood group A/B/H antigens and globo-series glycans were highly expressed in undifferentiated cell lines while colon-like cell lines showed a high abundance of (sialyl-)Lewis^A/X^, Lewis^B/Y^ antigens, and *α*1,3/4-fucosylation (Table 1). Especially, Lewis^B/Y^ was highly expressed in colon-like cell lines and significantly positively correlated with the gene expression of *FUT2*, which has previously shown to be increased in colon tumors (29). Moreover, our data revealed strong associations between (sialyl-)Lewis antigen expression and *FUT3* expression, which is one of GTs essential for the biosynthesis of sialyl-Lewis antigens (30). Specifically, (sialyl-)Lewis^A/X^ antigens were expressed in high abundance by the mucin-secreting cell lines LS180 and LS174T, which was consistent with the recent finding on *O*-glycans (20). Whereas, a higher amount of Lewis antigens on GSL glycans was also detected in SW1463 cell line, which was not the case in the *O*-glycome (20). In our study, we discovered significant correlations of (sialyl-)Lewis^A/X^ expression between the GSL glycome and *O*-glycome (supplemental Fig. S-5 and Table S-11), and similar correlations were found for the Lewis^A/X^ and ^B/Y^ antigens. Moreover, previous *N*-glycosylation studies of the same set of cell lines showed increased antenna fucosylation, particularly in the colon-like cell lines LS180, LS174T, HT29, WiDr, and SW1116, indicative of the presence of Lewis-type antigens (31). Previous studies have revealed that an increased expression of sialyl-Lewis^A/X^ antigens is associated with poor prognosis and sialyl-Lewis^X^ could be a potential prognostic marker in CRC (6, 7). This is in line with our finding that a high expression of sialyl-Lewis^A/X^ in colon-like cell lines belonged to either CMS2 or CMS3 (19), suggesting that sialyl-Lewis^A/X^ might be a marker for CMS2/3 CRC tumors.

In contrast, the undifferentiated cell lines were characterized by a high abundance of blood group A, B, and H antigens. These findings were consistent with the expression of blood group antigens in *O*-glycans (supplemental Fig. S-5) (20). The LOVO cell line obtained from an individual with blood type B expressed particularly high levels of blood group B antigens on the GSL glycans (32). Similarly, cell lines derived from patients with blood type A (SW480, SW620, and HT29) revealed a relatively high abundance of blood group A antigens on GSL glycans (20, 32). The Caco-2 cell line (O-blood group individual) expressed blood group H antigens for both GSL and *O*-linked glycans (20). Surprisingly, blood group A antigens were found in cell line SW1116, which is derived from an O-blood type individual; however, this is in agreement with previous findings (32). Interestingly, no blood group antigens on GSL glycans were detected for the LS174T cell line (individual with blood group O). In the SW48 cell line, derived from an individual with blood type AB, only blood group A antigens were found for both the GSLs and *O*-glycome (supplemental Table S-6) (20). Whilst cell lines HCT15, HCT8, and DLD-1 were obtained from the same patient, they showed profound and diverse glycosylation profiles: high expression of blood group H antigens was found in HCT15 and HCT8, while no expression of blood group H antigens were expressed in cell line DLD-1, which was consistent with the results studying the *O*-glycome (20). While good associations were found between the GSL and *O*-glycome, further studies are needed to investigate the *N*-glycome of these CRC cell lines. This can further confirm the specificity of expression of blood group antigens in certain CRC cell lines, especially for the undifferentiated cell lines, which correspond to CMS1/4 CRC tumors (19).

Increased sialylation of glycans is observed in various cancers including colon cancer, and especially, the *α*2,3-sialylation is suggested to play an essential role in metastasis with regards to EMT (4). Moreover, undifferentiated cell lines show an upregulation of transforming growth factor β–induced genes and EMT signatures (19). In our study, a high expression of (*α*2,3-)sialylation was found for the undifferentiated cell lines DLD-1 and HCT116 as well as colon-like cell line WiDr. The aberrant expression of sialylation may result from the dysregulation and/or altered activity of sialyltransferases. Compared to *α*2,3-sialylation, the *α*2,6-sialylation is critical for the malignant and metastatic progression, for which ST6GAL1 plays an important role (33, 34). This is supported by a study in which *ST6GAL1* knockdown resulted in a significant suppression in metastasis of the colon-like cell line HT29 (35). Moreover, the upregulation of *α*2,6-sialylation was found in colon cancer tissue (36). Correlation analysis showed that the *α*2,6-sialylation on GSL glycans had a strong association with *α*2,6-sialylation found on the *O*-glycome of CRC cell lines (supplemental Fig. S-5). In our study, a high expression *α*2,6-sialylation on GSL glycans was detected in cell lines SW1398, LS411N, and SW620, which can be selected as suitable models for studying the function of *α*2,6-sialylation and *ST6GAL1* during CRC invasion.

A previous study indicated that GCNT2 and its corresponding I-branched glycans accelerate EMT in CRC (37). I-branched glycans are considered as emerging effectors of malignant progression and were found to be relatively highly expressed in the undifferentiated cell lines SW873 and Caco-2 (33). Moreover, this feature correlated positively with *GCNT2* gene expression (Fig. 3). Neu5Gc is normally not found in humans attributable to a deletion of the gene *cmah* (38). Previous studies have indicated that the detection of Neu5Gc in human cells is most likely due to the dietary incorporation of glycoproteins carrying Neu5Gc (39). It is highly likely that the found expression of Neu5Gc in the CRC cell lines of this study is due to uptake and incorporation from the culture medium, which contains 10% FBS.

The findings in our study highlight the value of an in-depth glycomic study as different glycan motifs may present different functions. For example, it is known from previous studies that specific glycosylation features such as *α*2,3-sialylation and I-branching play an essential role in the EMT and *α*2,6-sialylation and sialyl-Lewis^A/X^ in the metastasis of CRC. Nevertheless, the specific mechanism of how cancer-associated GSL-glycome features are involved in the progress of CRC remains unknown. Therefore, specific CRC cell line models can be selected for future studies on the basis of their glycome.

To gain insights into the potential regulation of GSL glycans, a correlation study of GSL glycans with TFs was performed. Our study showed that high expression of (sialyl-)Lewis^A/X^, Lewis^B/Y^ antigens, and fucosylation (specifically *α*1,3/4-fucosylation) in colon-like cell lines were significantly correlated with expression of *FUT3* and presented positive correlations with TFs that are upregulated in colon-like cells including CDX1 (Fig. 3). CDX1 as an intestine-specific TF is involved in the regulation of different biological processes including proliferation, apoptosis, and cell adhesion and associated with oncogenesis of the gastrointestinal tract (40, 41). An association of highly fucosylated *N*-glycans with *CDX1* in CRC cell lines was revealed and validated by Holst *et al.*, with the finding of *CDX1* high-expressing CRC cell lines characterized by higher expression of antenna fucosylation (Lewis^A/X^ and ^B/Y^) on *N*-glycans (31, 41). Meanwhile, a positive correlation between *FUT3* and high expression of *CDX1* in CRC cell lines was discovered (41). Besides, previous studies indicated that TFs HNF1A and HNF4A associated with intestinal development (42) and played an essential role in the expression of antenna fucosylation *via* the regulation of *FUT3* (43), which are consistent with our findings of correlations between *FUT3* and *CDX1*, *HNF1A*, and *HNF4A* (supplemental Fig. S-4). Furthermore, undifferentiated cell line HCT116 lacked *CDX1* expression while showing more aggressive behavior. Interestingly, *CDX1* expression in HCT116 cells induced intestinal epithelial differentiation to crypt-forming colonies (44). Taking our findings and results from previous publications, we speculate that the aforementioned TFs, particularly CDX1, may play roles in the expression of Lewis-type glycosylation features *via* the upregulation of FUT3, which may contribute to the differentiation of CRC cell lines. Our findings pave the way for future functional studies validating the generated hypotheses of the regulation of GSL glycan expression in CRC cell lines.

## Conclusions

Our work revealed a strikingly diverse expression of GSL glycans in 22 CRC cell lines and provides an insight into the alteration of glycosylation during the development of CRC. The specific GSL glycome signatures were found for each CRC cell line classification. Additionally, correlations were found between GSL glycans and their corresponding GTs, which are known to be involved in the GSL biosynthesis providing valuable information on the regulation of GSL glycan expression in CRC cell lines. Besides, we hypothesize that certain TFs might play a role in the expression of fucosylated GSL glycans through the regulation of FUT3 on the basis of the discovered correlations between GSL glycans and selected GT and TF genes as well as between GTs and certain TFs. However, mechanistic studies are needed to confirm the role of specific TFs such as CDX1 in the regulation of GSL glycans expression. In conclusion, our in-depth analysis of GSL glycans profiling in CRC cell lines provides an informative resource for future research on biomarkers for CRC and paves the way for further studies on the regulation of GSL glycans in CRC.

## Data Availability

The raw mass spectrometric data files supporting the findings of this study are available in GlycoPOST in mzXML format, with the identifier GPST000239, accessible *via* the following link: https://glycopost.glycosmos.org/preview/164822151461d35202311d4 with pin code: 5211.

## Supplemental data

This article contains supplemental data (19, 20, 24, 26, 27, 45).

## Conflicts of interest

The authors declare no competing interests.
