# Supplementary figures and images for: High Diversity of Glycosphingolipid Glycans of Colorectal Cancer Cell Lines Reflects the Cellular Differentiation Phenotype
Source: Mol Cell Proteomics. 2022 Apr 28;21(6):100239. doi: 10.1016/j.mcpro.2022.100239 (PMC9157004; doi:10.1016/j.mcpro.2022.100239)

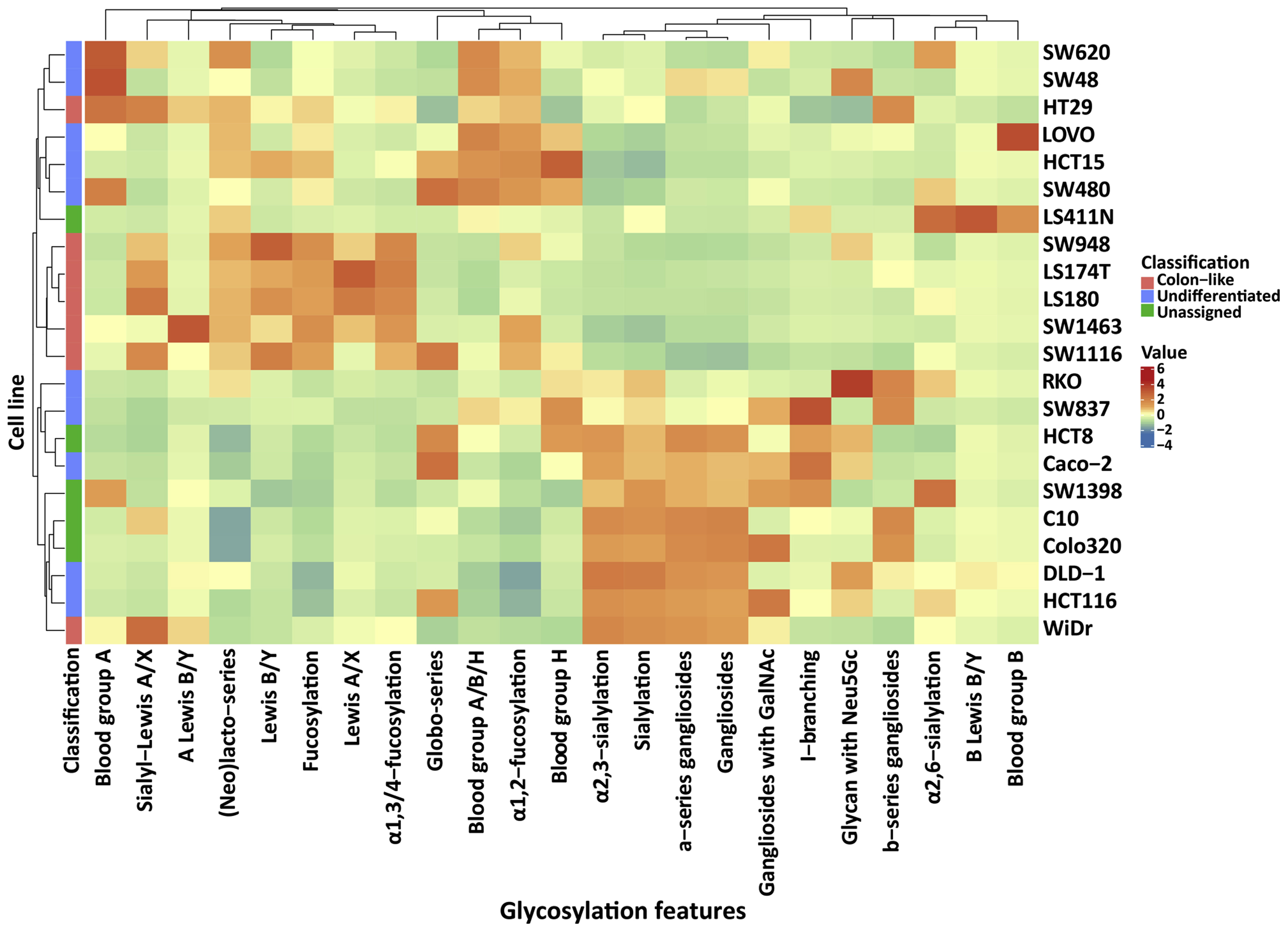

Supplement: Supplementary Figure S-1 [file figs1.jpg]

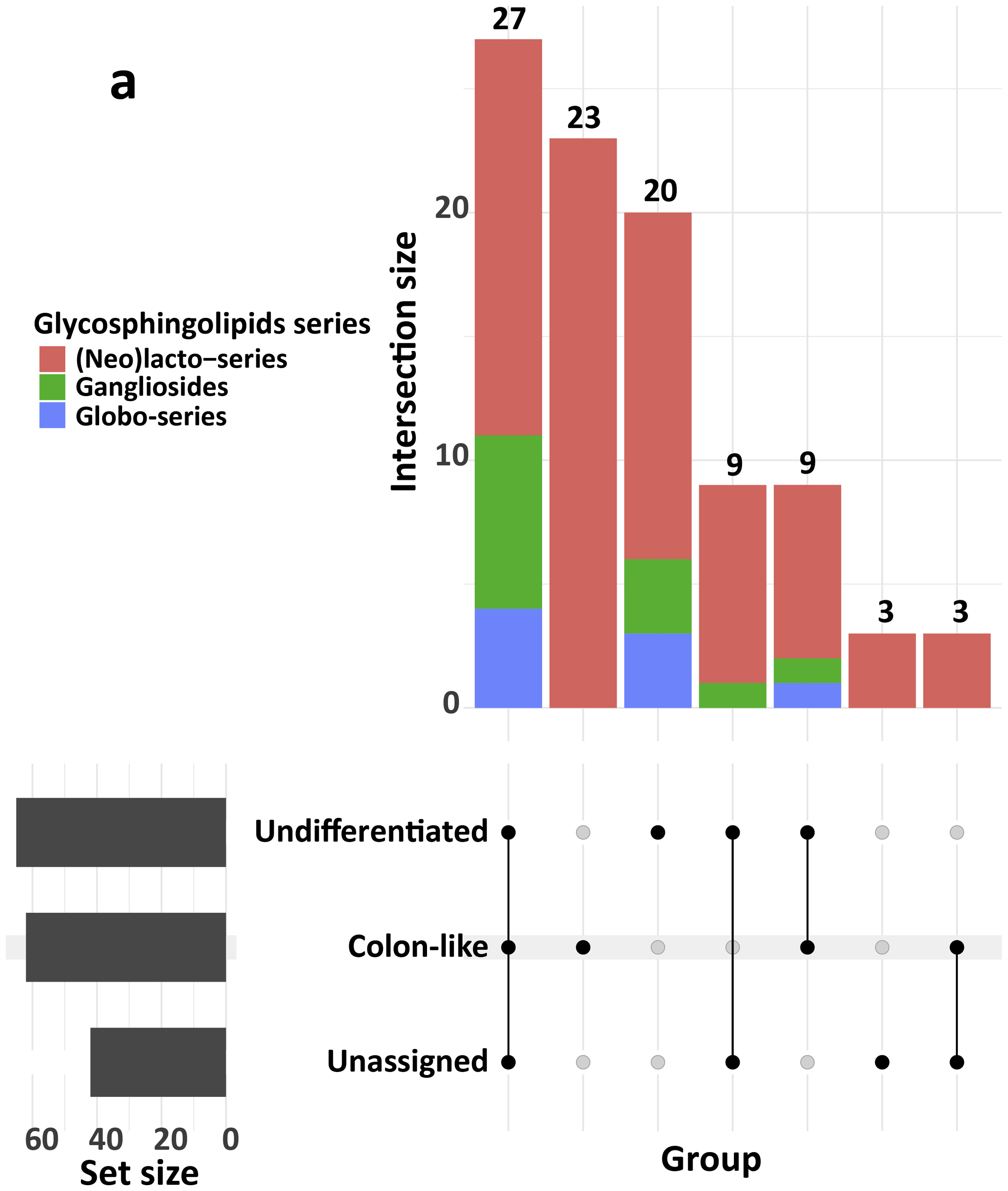

Supplement: Supplementary Figure S-2a [file figs2.jpg]

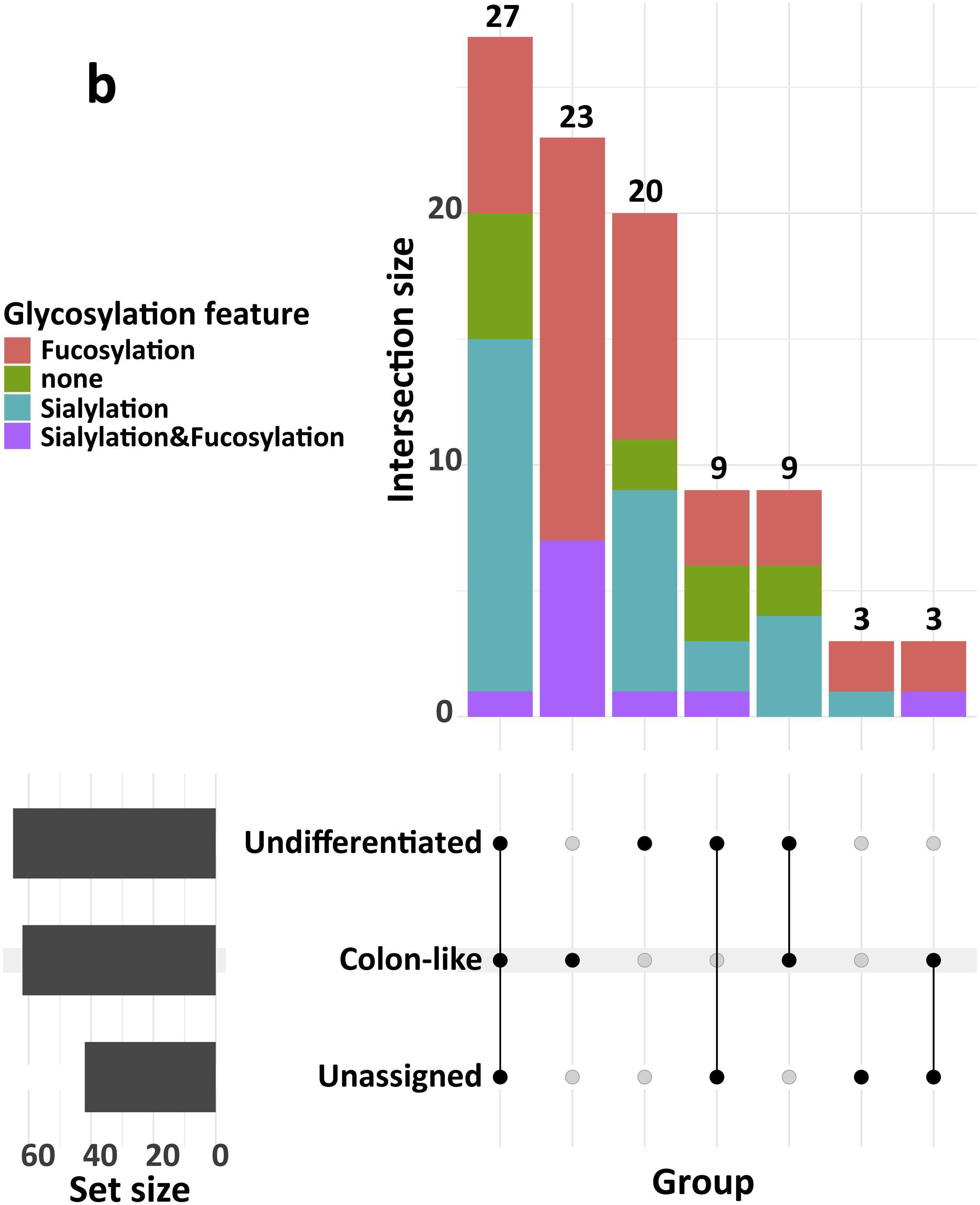

Supplement: Supplementary Figure S-2b [file figs3.jpg]

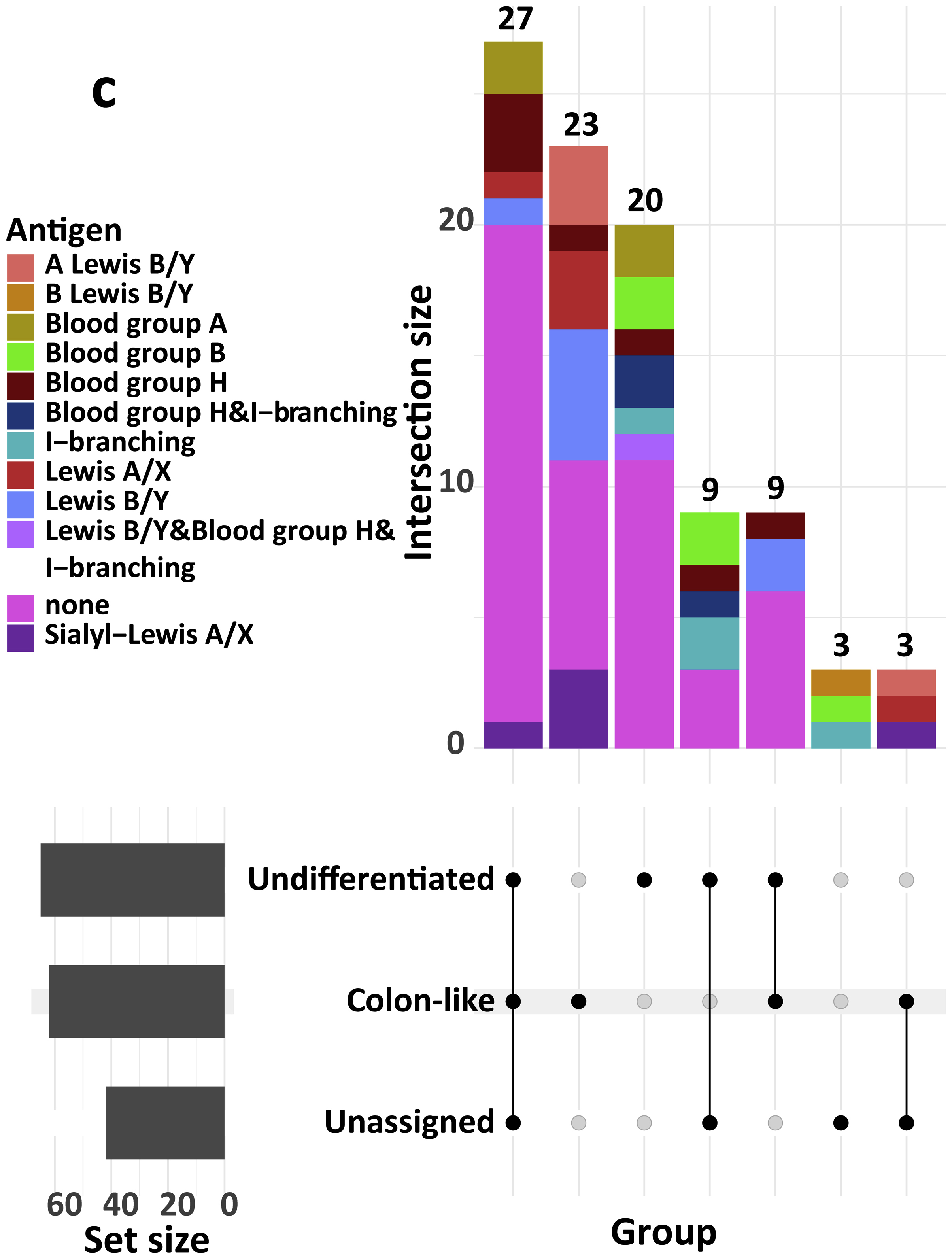

Supplement: Supplementary Figure S-2c [file figs4.jpg]

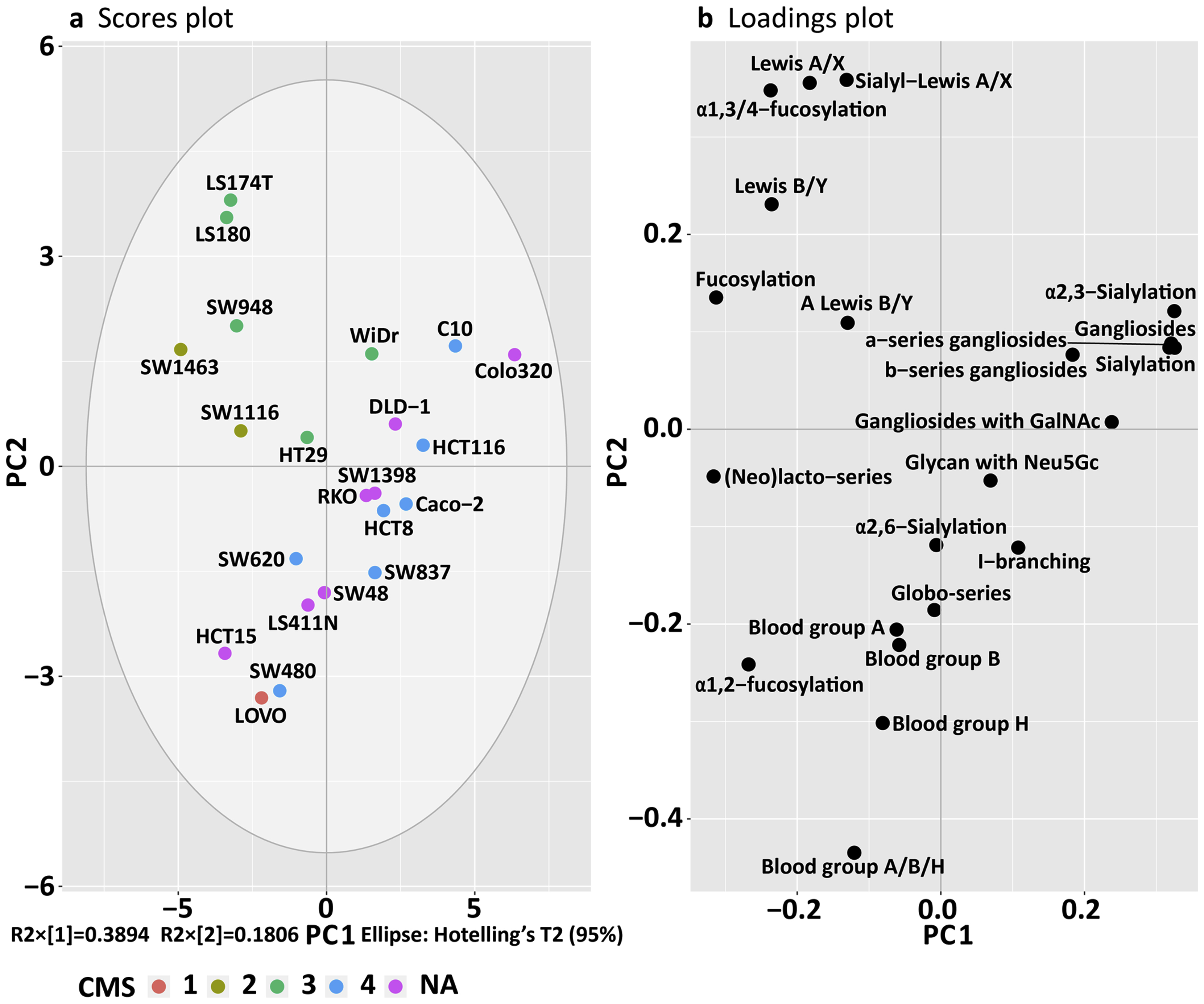

Supplement: Supplementary Figure S-3 [file figs5.jpg]

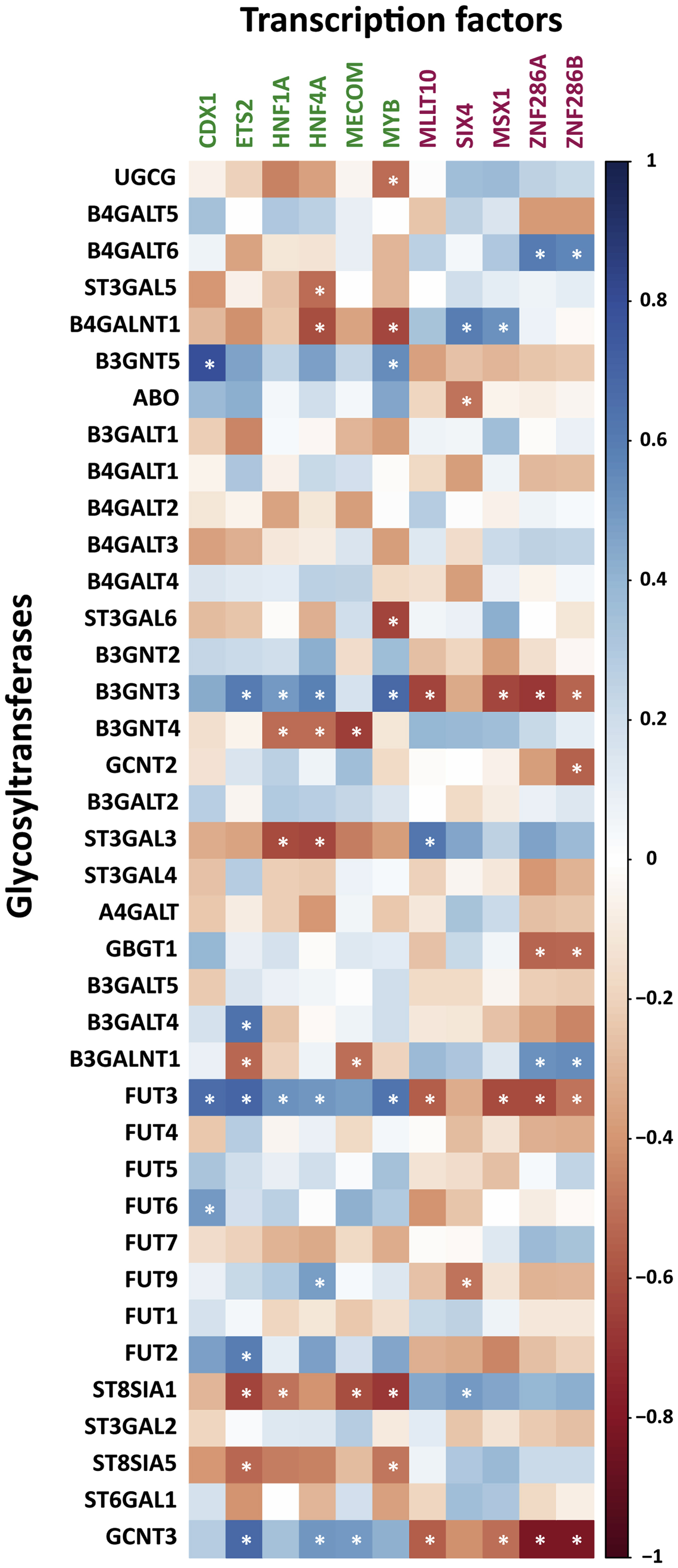

Supplement: Supplementary Figure S-4 [file figs6.jpg]

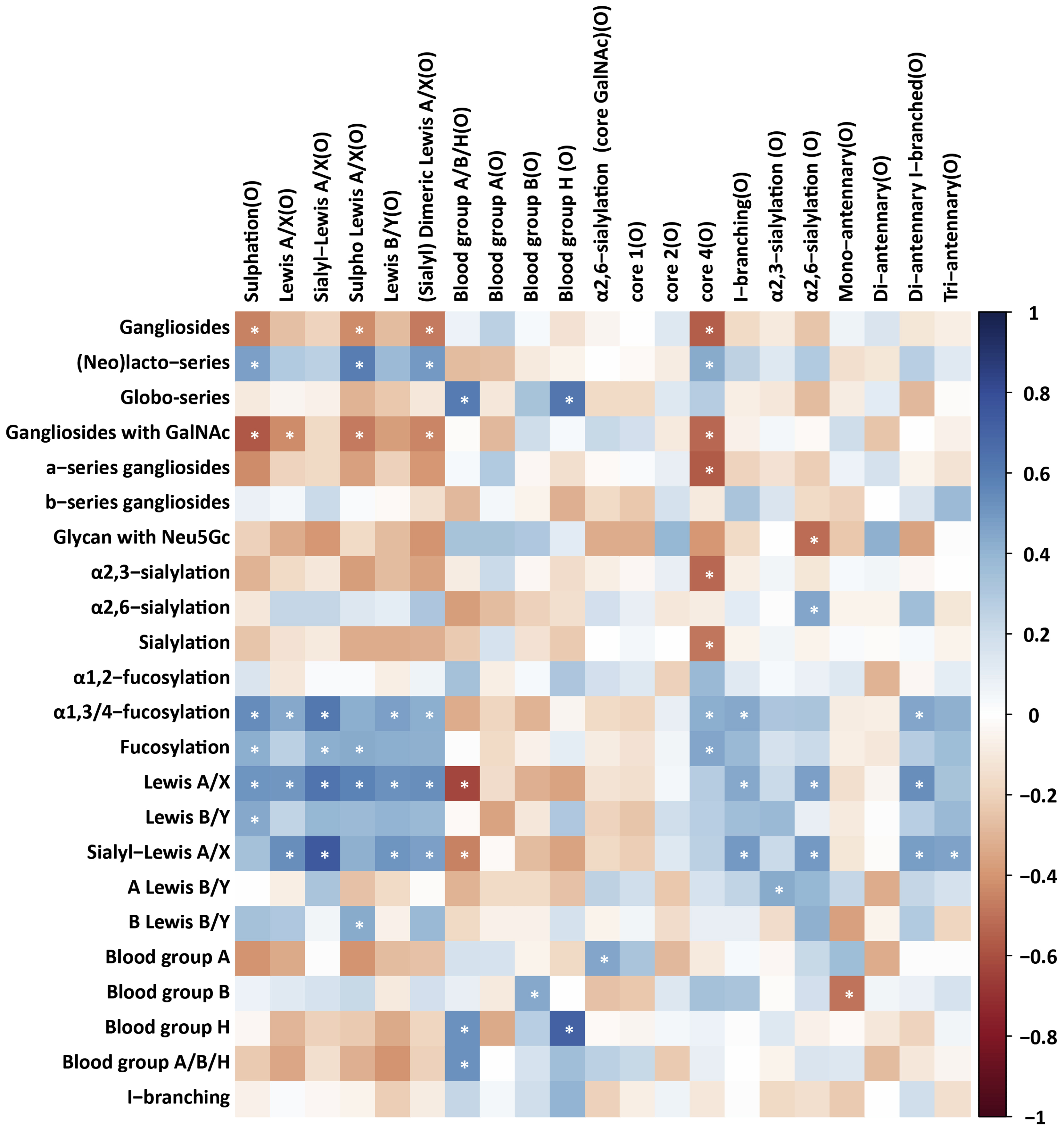

Supplement: Supplementary Figure S-5 [file figs7.jpg]
