# Supplementary material for: High Diversity of Glycosphingolipid Glycans of Colorectal Cancer Cell Lines Reflects the Cellular Differentiation Phenotype
Source: Mol Cell Proteomics. 2022 Apr 28;21(6):100239. doi: 10.1016/j.mcpro.2022.100239 (PMC9157004; doi:10.1016/j.mcpro.2022.100239)
Supplement: Supplementary Figures [file mmc1.pdf]

Supplementary Material

High diversity of glycosphingolipids glycans of colorectal cancer cell lines reflects the cellular differentiation phenotype

Di Wang [1], Katarina Madunić [1], Tao Zhang [1], Oleg A. Mayboroda [1], Guinevere S.M. Lageveen-Kammeijer [1], Manfred Wuhrer [1,*]

[1] Leiden University Medical Center, Center for Proteomics and Metabolomics, Postbus 9600, 2300 RC Leiden, The Netherlands

* Correspondence: m.wuhrer@lumc.nl, tel: +31(0)715266989

Table of contents

Figure S-1 2
Figure S-2 3
Figure S-3 4
Figure S-4 5
Figure S-5 6
References 7

Figure S-1

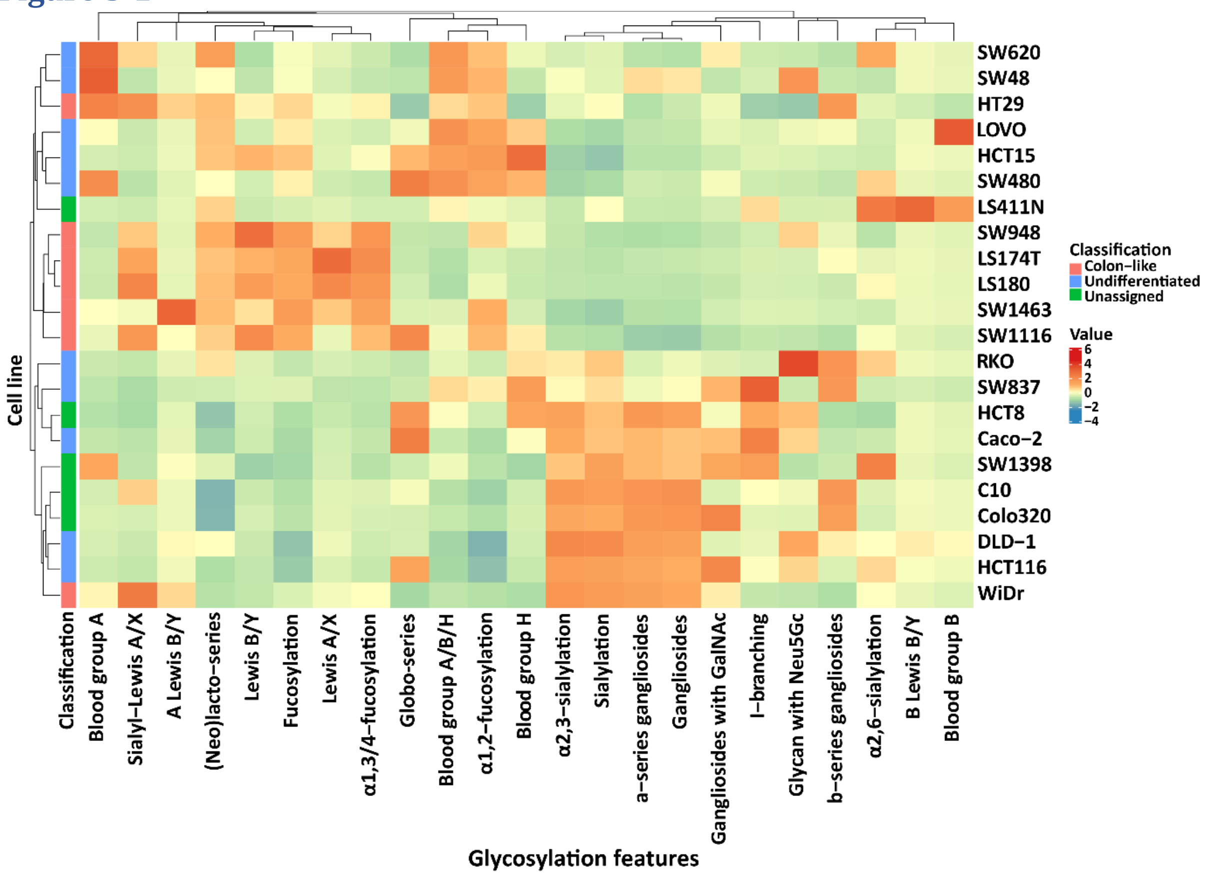

Supplementary Figure S-1. Distribution of GSL glycosylation features in 22 CRC cell lines. Relative quantification of glycosylation features (bottom) was calculated for each cell line (right) and presented in a clustered heatmap. The classification of the CRC cell lines is marked using color codes (colon-like in red, undifferentiated in blue and unassigned in green).

Figure S-2

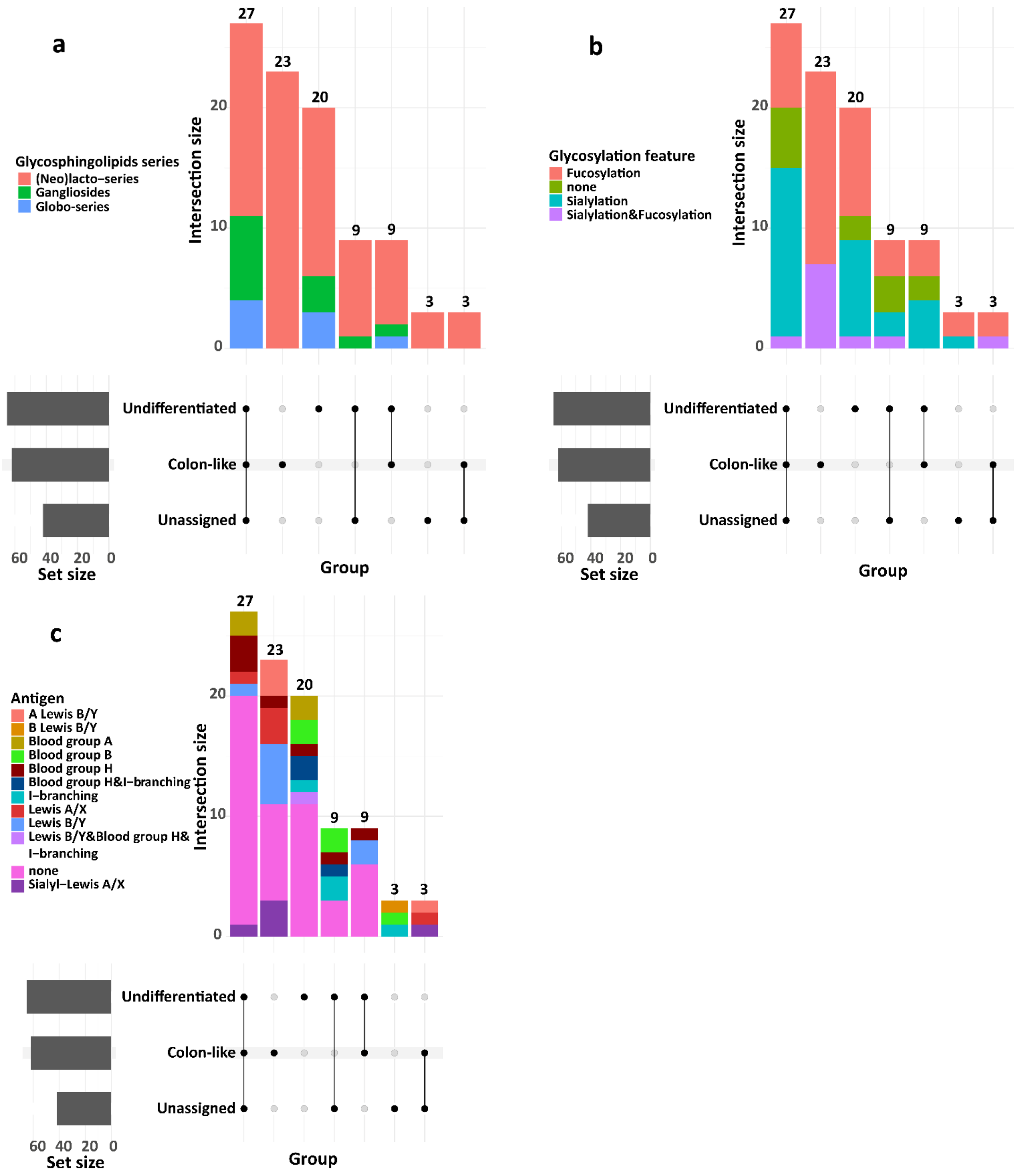

Supplementary Figure S-2. Expression of specific GSL glycans and glycan traits and their intersections in different CRC cell line classifications. The upset plot depicts the number of glycans detected specifically in each group (colon-like, undifferentiated, or unassigned) and their intersections indicating the number of shared glycans between the groups. Bars are colored by **(a)** glycosphingolipids series **(b)** glycosylation feature and **(c)** antigen expression, none means GSL glycans without any antigen on**.** Counts are displayed on top of each bar (**Supporting Information, Table S-8**).

Figure S-3

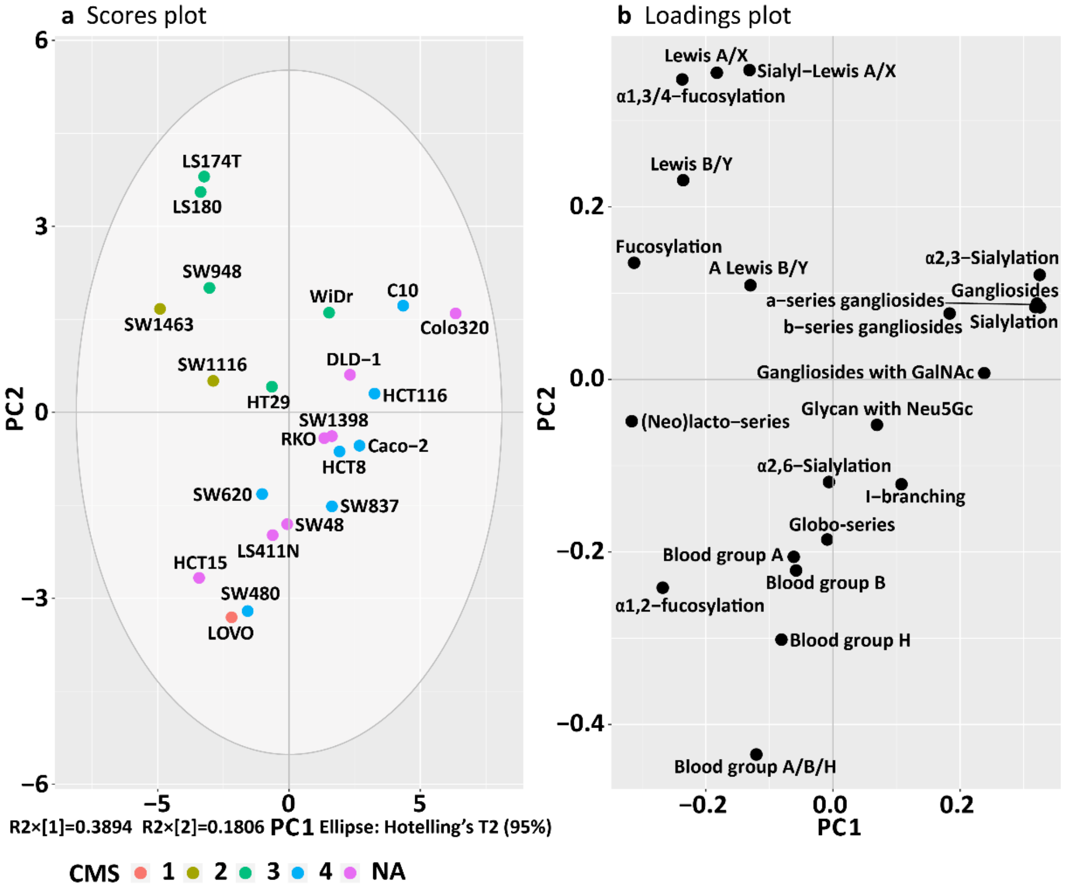

Supplementary Figure S-3. PCA of calculated GSL glycosylation features on the basis of relative quantification. (a) PCA scores plot of PC1 against PC2 indicates a dissociation of CRC cell lines belonging to different CMS classifications driven by the variables (glycosylation features) depicted in the **(b)** PCA loading plot. The top two principal components explain 57% of the variation within the data. Technical replicates (n = 2) were averaged per cell line.

Figure S-4

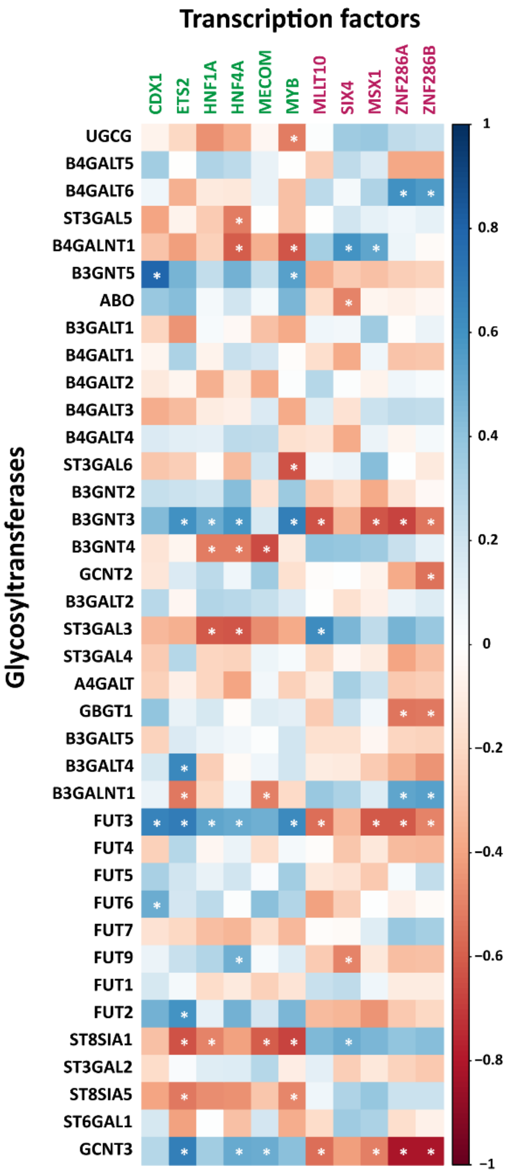

Supplementary Figure S-4. Correlation between gene expression of glycosyltransferases related to biosynthesis of GSL glycans with gene expression of transcription factors illustrated in a correlation matrix. A spearman correlation was performed with a significance cut-off of $p \leq 0.05$. * displays statistically significant correlations. The correlation coefficients are color-coded as indicated in the legend. The TFs in green indicate the high expression in colon-like cell lines. The TFs in dark pink present that the high abundance found in undifferentiated cell lines.

Figure S-5

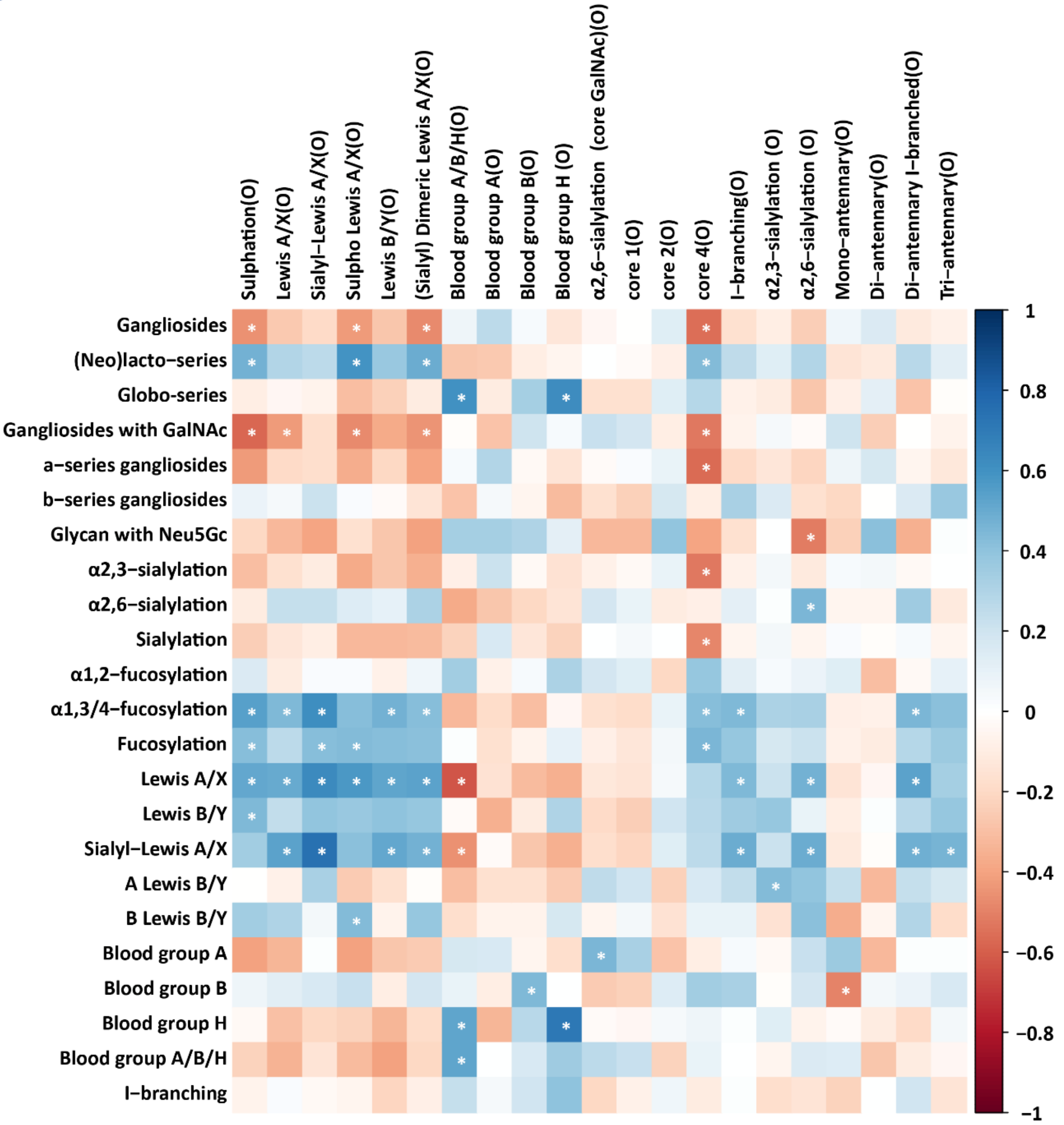

Supplementary Figure S-5. Correlation between GSL glycosylation features and *O*-glycosylation features are illustrated in a correlation matrix for CRC cell lines. A spearman correlation method was performed with a significance cut-off of $p \leq 0.05$. * indicates statistically significant correlations. The correlation coefficients are color-coded as indicated in the legend. The glycosylation features ending with (O) indicate *O*-glycosylation features which were retrieved from previous study [1].

References

1. Madunic, K., T. Zhang, O.A. Mayboroda, S. Holst, K. Stavenhagen, C. Jin, N.G. Karlsson, G.S.M. Lageveen-Kammeijer and M. Wuhrer (2021) *Colorectal cancer cell lines show striking diversity of their O-glycome reflecting the cellular differentiation phenotype*. Cell Mol Life Sci **78**(1): p. 337-350.https://doi.org/10.1007/s00018-020-03504-z
